# Levels of S100B protein drive the reparative process in acute muscle injury and muscular dystrophy

**DOI:** 10.1038/s41598-017-12880-9

**Published:** 2017-10-02

**Authors:** Francesca Riuzzi, Sara Beccafico, Roberta Sagheddu, Sara Chiappalupi, Ileana Giambanco, Oxana Bereshchenko, Carlo Riccardi, Guglielmo Sorci, Rosario Donato

**Affiliations:** 1 Department of Experimental Medicine, Perugia Medical School, University of Perugia, Piazza Lucio Severi 1, 06132 Perugia, Italy; 2 Department of Medicine, Perugia Medical School, University of Perugia, Piazza Lucio Severi 1, 06132 Perugia, Italy; 3 Istituto Interuniversitario di Miologia, Perugia Medical School, University of Perugia, Piazza Lucio Severi 1, 06132 Perugia, Italy; 4 Centro Universitario per la Ricerca sulla Genomica Funzionale, Perugia Medical School, University of Perugia, Piazza Lucio Severi 1, 06132 Perugia, Italy

## Abstract

Regeneration of injured skeletal muscles relies on a tightly controlled chain of cellular and molecular events. We show that appropriate levels of S100B protein are required for timely muscle regeneration after acute injury. S100B released from damaged myofibers and infiltrating macrophages expands the myoblast population, attracts macrophages and promotes their polarization into M2 (pro-regenerative) phenotype, and modulates collagen deposition, by interacting with RAGE (receptor for advanced glycation end-products) or FGFR1 (fibroblast growth factor receptor 1) depending on the muscle repair phase and local conditions. However, persistence of high S100B levels compromises the regeneration process prolonging myoblast proliferation and macrophage infiltration, delaying M1/M2 macrophage transition, and promoting deposition of fibrotic tissue via RAGE engagement. Interestingly, S100B is released in high abundance from degenerating muscles of *mdx* mice, an animal model of Duchenne muscular dystrophy (DMD), and blocking S100B ameliorates histopathology. Thus, levels of S100B differentially affect skeletal muscle repair upon acute injury and in the context of muscular dystrophy, and S100B might be regarded as a potential molecular target in DMD.

## Introduction

Upon injury skeletal muscles initiate a repair process leading to tissue regeneration. Central to muscle regeneration are adult muscle stem cells known as satellite cells (SCs)^1^, with the participation of other cell types such as vascular pericytes^2,3^ and fibro/adipogenic precursors^4,5^. Molecules passively released from damaged muscle tissue or secreted by infiltrating immune cells give rise to a complex tissue response; SCs exit their quiescent state, proliferate, migrate and differentiate into fusion competent myocytes that eventually fuse with damaged myofibers to repair them and form new myofibers. The regulation of SC proliferation and differentiation relies in part on the activity of extracellular factors (i.e. hormones, growth factors, cytokines and components of the extracellular matrix) and danger-associated molecular patterns (DAMPs)^1,6–8^ such as high mobility group box 1 protein (HMGB1) released from damaged muscle tissue^9,10^. Extracellular signals act via cell surface receptors responsible for the activation of intracellular signaling pathways leading to the coordinated expression and/or activation in myoblasts of the transcription factors, PAX7, MyoD, Myf5 and myogenin, which ultimately drive muscle regeneration.

Macrophages infiltrating acutely injured muscles play a prominent role in muscle regeneration, with an early transition from a proinflammatory (M1) phenotype (the dominant phenotype during the first 3 days post-injury) to an antiinflammatory (M2) phenotype (during the subsequent 5 days) being crucial for efficient tissue repair^7,11,12^. Interferon (IFN)-γ, interleukin (IL)-6 and tumor necrosis factor (TNF)-α are responsible for the expression of CD68 and inducible nitric oxide synthase (iNOS) in M1 (classically activated) macrophages that exert proinflammatory, phagocytic and cytolytic effects and stimulate myoblast proliferation, whereas IL-4 and IL-10 are responsible for the expression of CD163a and arginase-1 in M2 (alternatively activated) macrophages that exert antiinflammatory effects and promote muscle regeneration^13^. Whether extracellular factors other than cytokines intervene in the skewing of macrophage from M1 to M2 phenotype is incompletely understood. However, in a chronic muscle disease setting such as Duchenne muscular dystrophy (DMD) unrestricted liberation of DAMPs from damaged myofibers fuels infiltration with M1 macrophages, which leads to persistent degeneration/regeneration cycles causing progressive depletion of the muscle stem cell pool, chronic inflammation and fibrosis^14^.

S100B, a member of the S100 family of Ca^2+^-binding proteins of the EF-hand type, is expressed in mature myofibers and SCs^15,16^ and exerts intracellular and extracellular regulatory activities^17^. Extracellular S100B modulates myoblast differentiation^18^, and stimulates myoblast proliferation and reduces myoblast apoptosis *in vitro* by engaging its canonical receptor, the multiligand receptor for advanced glycation endproducts (RAGE, encoded by *Ager*), or enhancing bFGF-FGFR1 signaling depending on myoblast density^19–21^. Early upon acute injury muscles release S100B in advance of bFGF and HMGB1 with declining release thereafter^21^, suggesting that S100B might regulate the initial phases of the regeneration process. Whether S100B released in damaged muscles participates in the reparative process and/or the pathophysiology of muscle diseases is not known.

We show here that: 1) early after acute muscle injury released S100B expands the myoblast population, attracts macrophages to damage sites and promotes M1/M2 macrophage switch in a RAGE-dependent manner; 2) during the M2 macrophage phase S100B promotes M1/M2 macrophage switch via stimulation of RAGE and/or FGFR1 activity depending on local conditions; 3) infiltrating macrophages transiently express and release S100B that contributes to the regeneration process together with myofiber-released S100B; and 4) if present in high abundance at damage sites (e.g. in muscular dystrophy) S100B sustains the M1 macrophage phase via excess RAGE engagement thereby compromising regeneration and promoting fibrotic tissue deposition. Thus, levels of S100B dictate its effects in acute and persistent muscle injury.

## Materials and Methods

### Protein purification, myoblast culture and characterization of anti-S100B neutralizing antibody

S100B and S100A1 were purified as described^22,23^. S100B was made free of contaminating bacterial endotoxin as described^24^. S100B and S100A1 were run on SDS polyacrylamide gels (15%) and either stained with blue Coomassie or transferred onto nitrocellulose paper for western blotting using a polyclonal anti-S100B antibody (Abcam No. ab41548).

C2C12 myoblasts were seeded in 24-multiwell plates at 5 × 10^4^ density in DMEM (Invitrogen) supplemented with 20% fetal bovine serum (FBS) (Invitrogen), 100 U/ml penicillin, and 100 mg/ml streptomycin (growth medium), in an H_2_O-saturated 5% CO_2_ atmosphere at 37 °C. After 24 h myoblasts were shifted to DMEM containing 2% horse serum (Euroclone) and antibiotics as above (differentiation medium) for 2 days in the absence or presence of 200 ng S100B/ml ± an S100B neutralizing antibody (Abcam No. ab41548) or non-immune IgG (Sigma) (100 ng/ml). Cell lysates were subjected to western blotting for detection of eMyHC.

In other experiments C2C12 myoblasts were seeded as above on 13 mm-diameter coverslips. After 24 h myoblasts were shifted to conditioned medium (CM) obtained from peritoneal macrophages cultured in DMEM containing 0.25% horse serum plus or minus either IFN-γ or IL-10. CM was preincubated with neutralizing anti-S100B antibody or non-immune IgG (Sigma) (100 ng/ml) for 2 h before treatment of myoblasts. After 24 h myoblast proliferation was measured by bromodeoxyuridine (BrdU) incorporation assay, and myoblast differentiation was analyzed by myogenin expression. BrdU (Sigma) was added to cultures 2 h before fixation with cold methanol at 22 °C and processing by immunofluorescence using a monoclonal anti-BrdU antibody (1:50, Santa Cruz Biotechnology). Nuclei were counterstained with DAPI. BrdU^+^ and total cells were counted. Myogenin ﻿was detected by western blotting.

### Antibodies and oligonucleotide sequences

Antibodies and oligonucleotide sequences are listed in Supplementary Tables 1–3.

### Animals and experimental muscle injury

*In vivo* studies were performed on male WT (C57BL/10; original breeding from The Jackson Laboratory), *Ager* ^−/−^ (C57BL/6; obtained from Angelika Bierhaus, Heidelberg, Germany), *mdx* (C57BL/10ScSn-Dmd^mdx^/J; original breeding from The Jackson Laboratory) and C57BL/6 (Charles River) mice. Muscle injury was performed by injection of 50 µl of an aqueous 1.2% (w/v) BaCl_2_ solution in TA muscle of 8-wk old wild-type and *Ager* ^−/−^ mice, under zolazepam/tiletamine anesthesia. At d1 or d4 p.i., TA muscles were injected with either a neutralizing rabbit polyclonal anti-S100B antibody (Abcam No. ab41548) or rabbit non-immune IgG (Sigma) (100 ng/muscle). TA muscles were removed at intervals, fixed in 4% formalin in PBS (pH 7.2) and paraffin embedded for histological and immunohistochemical and immunofluorescence analyses. In some experiments the FGFR1 inhibitor SU5402 (Sigma, 25 mg/kg) or vehicle was i.p. injected into injured mice. Approval of use of animals was obtained by the Ethics Committee of the Perugia University and the Ministero della Salute, Italy. All methods were performed in accordance with the relevant guidelines and regulations of the Perugia University and the Ministero della Salute, Italy.

### Histology

Four-µm muscle cross sections were stained with H&E. To quantify the extent of muscle regeneration, sections at 100 µm intervals for each muscle were analyzed and the total number of centrally nucleated myofibers per section was manually counted by three independent operators. Cross-sectional area analysis was performed on a total of 3000–4000 myofibers/experimental condition. TA muscles were cut crosswise from the upper, middle, and lower regions of the muscle (three sections per region; n = 6 animals per group). Slices were analyzed and photographed with a bright field microscope (Olympus BX51) equipped with a digital camera. Myofiber cross-sectional area was measured using Cell P Analysis Imaging Processing Olympus software. Interstitial cells were counted within the muscle tissue from the upper, middle, and lower regions of the muscle (three sections per region; n = 6 animals per group).

### Immunohistochemistry and immunofluorescence

Paraffin sections of control or treated TA muscles were cut at 4 μm, deparaffinized with xylene and rehydrated in a graded ethanol series. Antigen retrieval was performed by boiling for 1.5 h in 10 mM citric acid buffer (pH 6.0), and depletion of endogenous peroxidase was accomplished by treatment with 3% H_2_O_2_. The sections were probed with the primary antibodies listed in Supplementary Table 1. Primary antibodies were diluted (1:50) in blocking buffer [BB, TBS containing 0.01% Tween-20 (T-TBS) and 10% HS] and the sections incubated overnight in a humid chamber at 4 °C. After several washings with T-TBS, the sections were incubated with appropriate biotinylated antibodies (1:500 dilution; Vector Laboratories) for 1 h in BB. For MAC3 and RAGE staining horseradish peroxidase (HRP)-conjugated secondary antibodies were used (Santa Cruz Biotechnology). To assay muscle fiber injury the sections were labeled with biotinylated-conjugated mouse anti-IgG (1:500 dilution; Vector Laboratories) overnight in BB. The sections were then rinsed with T-TBS, incubated for 45 min with Vectastain ABC reagents (Vector Laboratories), washed again with TBS, and incubated with 0.01% 3,3-diaminobenzidine tetrahydrochloride (DAB), 0.006% H_2_O_2_ in 50 mM Tris-HCl (pH 7.4). Nuclei were counterstained with haematoxylin. The sections were then dehydrated and mounted with EuKitt mounting medium (Electron Microscopy Sciences). Slices were analyzed and photographed in a bright field microscope (Olympus BX51) equipped with a digital camera. The number of positive cells was counted on at least four transversal sections obtained at two different locations for each muscle (n = 6).

Double immunofluorescence reactions on tissue slices were performed as above except that PBS, pH 7.4, instead of TBS and a different BB (i.e., 1% glycine and 3% BSA in PBS) and buffer for antibody dilution (3% BSA in PBS) were used. The primary and secondary fluorophore-antibodies are reported in Supplementary Table 1. Nuclei were counterstained with DAPI. After rinsing, samples were mounted with fluorescent mounting medium (Dako Corporation) and viewed in an epifluorescence microscope (Leica DMRB) equipped with a digital camera.

### Macrophage isolation and treatment

C57BL/6 J mice received an i.p. injection of 4% sterile thioglycollate (Sigma-Aldrich) and were sacrificed 3 days later for peritoneal macrophage isolation as described^25^. Peritoneal macrophages were seeded at 1.0 × 10^6^ cells/well for 3 h in 24-well plates with complete DMEM, washed with PBS to remove cellular debris, and stimulated for 24 h in DMEM 0.25% FBS with IL-10 (10 ng/ml, Millipore) or IL-4 (20 ng/ml, Peprotech) to induce the M2 phenotype or IFN-γ (10 ng/ml, Millipore) to induce the M1 phenotype plus or minus other additions as indicated in figure legends. After stimulation cells were lysed in TRIsure™ (Bioline) for quantitative real-time PCR analyses. The CM obtained from peritoneal macrophages cultured as above, were trichloroacetic acid-precipitated for detection of S100B by western blotting.

To isolate macrophages from muscles, injured wild-type and *Ager* ^−/−^ TA muscles treated with either anti-S100B or non-immune IgG were dissected and cleaned of discernible fat, rinsed with fresh PBS and weighed. The muscles were minced to a fine pulp with surgical scissors in cold PBS and then transferred to centrifuge tubes containing 10 ml per gram muscle mass of collagenase Type IV (Sigma) (20 mg/ml) in DMEM. Supplemental Information for more details. Tubes were incubated at 37 °C for two 45 min periods, after which the suspension was aspirated, centrifuged at 850 × *g* and resuspended in PBS. The cell suspensions were filtered through a 70-mm cell strainer (Falcon) and centrifuged at 850 × *g* for 5 min. The filtered cells were applied to Histopaque 1077 (Sigma-Aldrich), collected from the Histopaque and DMEM interface, washed with complete DMEM and counted. Irrespective of the source, the macrophage-enriched fraction of mononuclear cells isolated by Histopaque 1077 was seeded onto plastic culture dishes. After 2 h the supernatant containing floating cells was discarded and adherent cells (i.e. macrophages – see Fig. S2c) were lysed for real-time PCR and western blot analyses. This same procedure was employed to analyze S100B and/or cytokine effects on isolated macrophages.

The purity of the macrophage preparations was assayed by indirect immunofluorescence of 2 × 10^5^ peritoneal or muscle-derived cells that were cultured on coverslips and immunolabeled with rat anti-MAC3 antibody (Sigma-Aldrich) followed by an TRITC-conjugated second antibody (BD Biosciences). Cells were visualized by fluorescence microscopy and macrophage purity was expressed as the percentage of total cells that were MAC3 positive.

### Western blotting

Muscle tissue was homogenized in 50 mM Tris pH 7.4, 150 mM NaCl, 1% Triton X-100, in the presence of a mixture of protease inhibitors (Roche Applied Science). The amount of protein in each samples was determined by Bradford assay and equal amounts of protein were size-separated by SDS-PAGE. The primary antibodies used in immunoblot analyses are listed in Supplementary Table 2. After incubation with the appropriate HRP-conjugated secondary antibodies (Santa Cruz Biotechnology), the immune reaction was developed by enhanced chemiluminescence (SuperSignal West Pico, Thermo Scientific). C-DiGit Blot Scanner (LI-COR, USA) was used for blot analysis.

### Real-time PCR

Total RNA was extracted from TA muscles or macrophages using TRIsure™ (Bioline) according to the manufacturer’s instructions and reverse-transcribed with M-MLV Reverse Transcriptase (Life Technologies). Real-time PCR analysis of mRNA contents was performed on Stratagene Mx3000 P (Agilent Technologies, USA) using HOT FIREPol EvaGreen qPCR Mix Plus (ROX) ready-to-use solution (Solis BioDyne) in the presence of the primer sets in Supplementary Table 3. *Gapdh* was used as an internal standard.

### Migration assay

For migration assay, we used Boyden chambers (pore size, 8 μm) (BD Biosciences). Primary myoblasts or macrophages were seeded (5.0 × 10^4^ cells) in DMEM in the upper chamber, and DMEM plus S100B (0–40 µg/ml) was placed in the lower chamber. After 20 h, the cells migrated to the lower side of the chamber were fixed in methanol, stained with crystal violet, and counted under a microscope (10 randomly chosen fields per sample).

### Macrophage depletion

Clodronate liposomes or empty liposomes (FormuMax) were i.p. injected (0.2 ml/mouse) into C57/BL6 mice 1 day before muscle injury. Anti-S100B antibody or non-immune IgG was injected into injured TA muscles at d1 p.i.

### FACS analyses

Staining with directly conjugated surface antibodies was performed as described^26^. The following antibodies were used: anti-CD11b (clone M1/70, eBioscience), anti-F4/80 (clone BM8, eBioscience), anti-FGFR1 (clone M19B2, eBioscience). Staining with unconjugated anti-CD163 antibody (Bioss) was revealed by anti-rabbit Alexa-647-conjugated secondary antibody (Invitrogen). Intracellular staining for iNOS was performed using the Foxp3/Transcription Factor Staining Buffer Set (Tonbo Bioscience). Analysis was done using a two-laser standard configuration Attune NxT Acoustic Focusing Cytometer (Invitrogen). Analysis of flow cytometry data was performed with FlowJo software (Tree Star Inc).

### *In situ* Proximity Ligation Assay

Macrophages isolated from injured *Ager* ^−/−^ TA at d4 p.i. were incubated for 30 min with 200 ng/ml S100B or vehicle. Cells were then fixed as described^21^, treated with a mixture of anti-S100B and anti-FGFR1 and subjected to *in situ* Proximity Ligation Assay (OLINK Bioscience, Uppsala) according to the manufacturer’s instructions. In control experiments anti-S100B antibody was omitted.

### Immunoprecipitation

Immunoprecipitation analysis of macrophages was performed as described^20^. Conditions were as for *in situ* proximity ligation assay except that isolated macrophages, following treatment for 30 min with 200 ng/ml S100B or vehicle, were subjected to immunoprecipitation with anti-S100B antibody (Abcam No. ab41548; 1 µg/ml). Immunoprecipitates were then probed with anti-S100B and anti-FGFR1 antibodies.

### Statistical analysis

Each experiment was repeated at least three times. Representative experiments are shown unless stated otherwise. The data were subjected to analysis of variance (ANOVA) with SNK post hoc analysis using a statistical software package (GraphPad Prism version 4.00, GraphPad). Data are the results of at least three independent experiments and are expressed as mean ± SEM.

## Results

### Blocking S100B early after acute muscle injury delays regeneration

**Figure 1:**
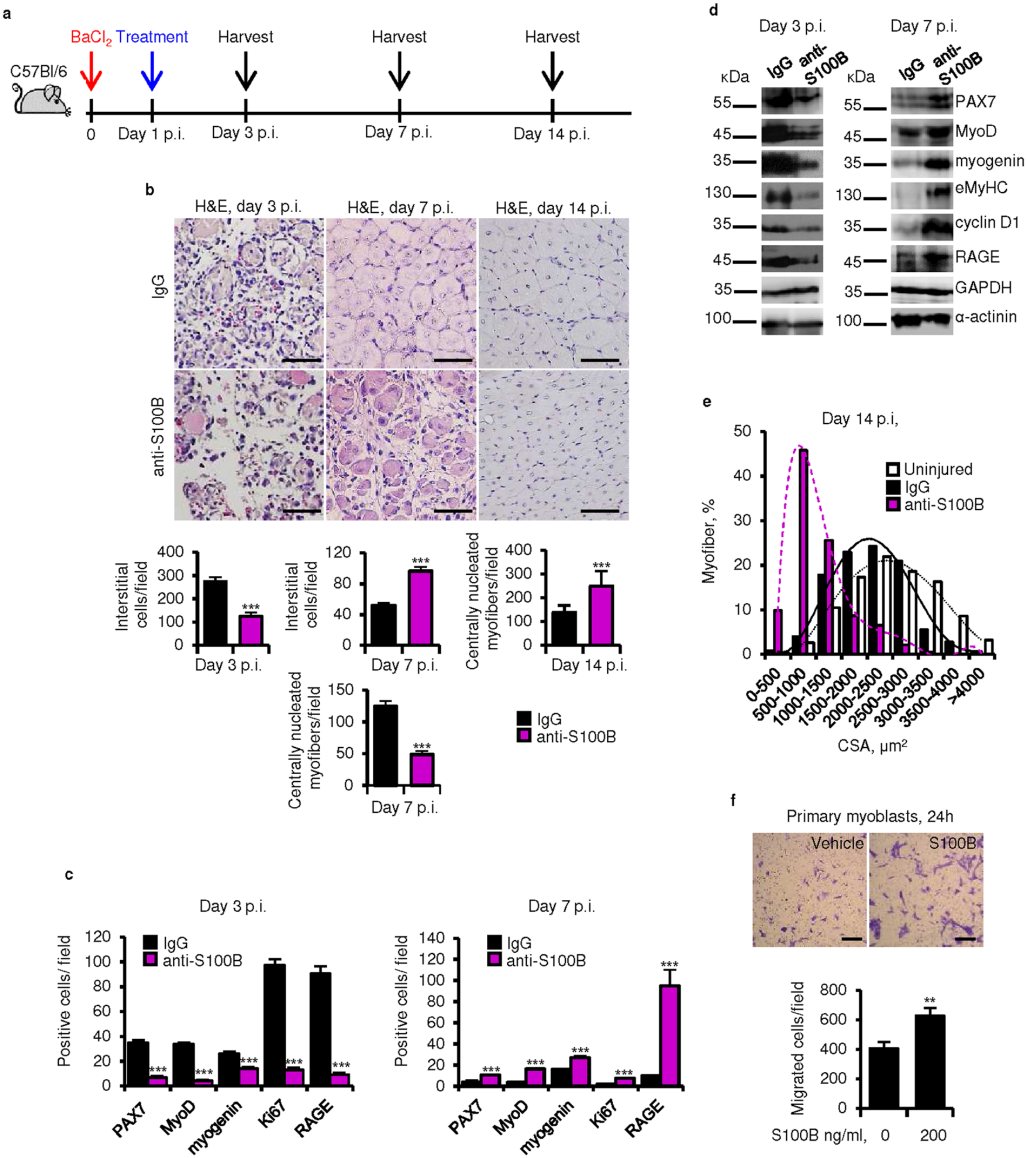
Blocking S100B early after acute muscle injury delays regeneration. (**a**) BaCl_2_-injured TA muscles were injected with IgG or a polyclonal anti-S100B antibody (Abcam No. ab41548) at d1 p.i. Treated muscles were excised at d3, d7 or d14 p.i. (**b**) Histology of muscles and counts of interstitial cells and centrally nucleated myofibers. (**c**) Counts of PAX7^+^, MyoD^+^, myogenin^+^, Ki67^+^, and RAGE^+^ cells (also see Fig. S1d,e). (**d**) Western blots of the indicated proteins in homogenates of IgG- and anti-S100B-treated muscles. Immunoblots of GAPDH and α-actinin are included as loading controls. (**e**) Myofiber size distribution at d14 p.i. in uninjured muscles and IgG- and anti-S100B-treated injured muscles. (**f**) Migration of primary mouse myoblasts in Boyden chambers in the absence or presence of S100B. Full-length blots are presented in Supplementary Fig. S7 “Fig. 1”. Results are means ± SEM (n = 6). **p < 0.01, ***p < 0.001 vs. control. The scale bar represents 50 µm in (**b**) and 200 µm in (**f**).

Acutely injured muscle tissue releases S100B [~20 ng/*Tibialis anterior* (TA) mouse muscle] at day 1 (d1) post-injury (p.i.) with declining release thereafter^21^. To investigate whether released S100B regulates muscle regeneration we injected an S100B-neutralizing antibody (100 ng/muscle) into BaCl_2_ damaged TA muscles at d1 p.i. and examined them at intervals (Fig. 1a). Preliminarily we found that (1) the S100B neutralizing antibody used throughout was specific to S100B (Fig. S1a); (2) anti-S100B antibody reduced S100B’s ability to inhibit the expression of the late myogenic differentiation marker, myosin heavy chain (MyHC)^18^ (Fig. S1b); and (3) when added to myoblasts cultured in differentiation medium without added S100B, this antibody stimulated MyHC expression consequent to neutralization of the S100B normally present in FBS^18^ (Fig. S1b). As investigated at d3 p.i., blocking S100B caused a significant decrease in the number of interstitial cells compared to non-immune IgG-injected muscles (Fig. 1b) partly due to decreased numbers of proliferating (Ki67^+^) cells (Fig. 1c and S1d). Experiments in which the S100B neutralizing antibody had been injected into the peritoneum at a dose of 1000 ng/mouse yielded identical results to those in Fig. 1 (results not shown).

In control injured muscles a fraction of interstitial cells were proliferating (PAX7^+^) and/or differentiating (MyoD^+^) myoblasts and differentiated (myogenin^+^) myocytes as expected; blocking S100B caused a significant decrease in the number of all these cell phenotypes (Fig. 1c). Blocking S100B also reduced the number of RAGE^+^ cells (Fig. 1c and S1d). RAGE becomes transiently expressed in activated SCs and proliferating/differentiating myoblasts^9^ and activated macrophages^27^, and RAGE ligands, including S100B, upregulate RAGE expression^27,28^. Western blot analysis of muscle tissue homogenates at d3 p.i. affirmed the decrease in levels of PAX7, MyoD, myogenin, embryonic MyHC (eMyHC), cyclin D1 (a proliferation marker) and RAGE in anti-S100B-treated muscles compared to controls (Fig. 1d). At d7 p.i. larger numbers of interstitial myoblasts, myocytes and Ki67^+^ cells and larger amounts of myogenic and proliferation markers were measured in anti-S100B-treated muscles (Fig. 1c,d and S1e) indicative of delayed regeneration. Consistently, compared to controls, in anti-S100B-treated muscles lower and higher numbers of centrally nucleated (regenerating) myofibers were counted at d7 and d14 p.i., respectively (Fig. 1b), with a higher density of thin myofibers at d14 (Fig. 1e). S100B also stimulated primary myoblast migration (Fig. 1f), pointing to a chemokinetic effect on myoblasts. Thus, during the first 3 days p.i. S100B might favor muscle regeneration by promoting myoblast proliferation and migration.

### S100B affects macrophages in acutely injured muscles

**Figure 2:**
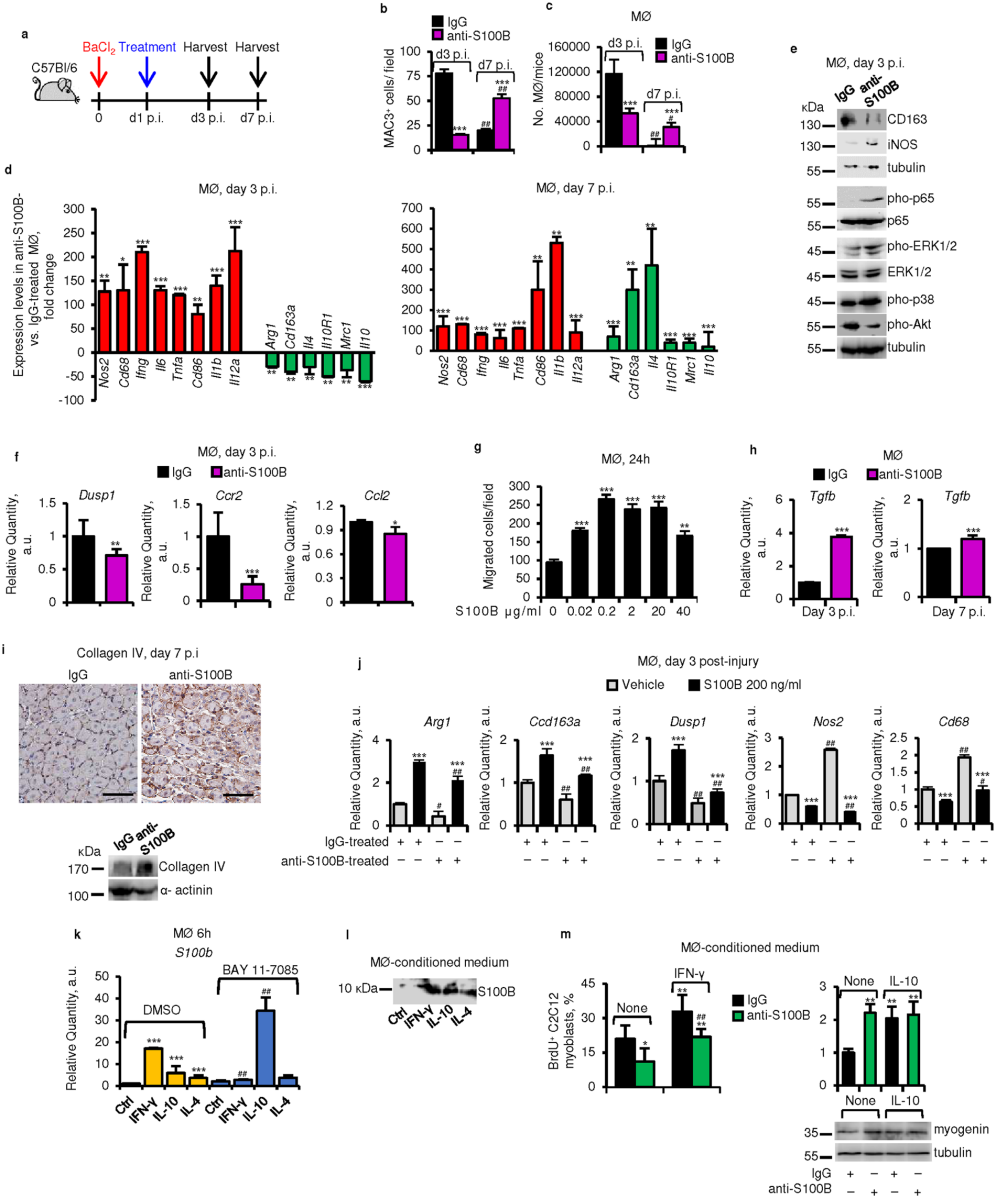
S100B affects macrophages early after acute muscle injury. (**a**) TA muscles were treated as described in the legend to Fig. 1a. Muscles were excised at d3 or d7 p.i. (**b**) Counts of MAC3^+^ cells. (**c**–**f,h**) Macrophages were isolated from IgG- and anti-S100B-treated injured muscles and either counted (**c**), analyzed by real-time PCR (**d**,**f**,**h**), or subjected to western blotting (**e**) (also see Fig. S2c). (**g**) Peritoneal macrophages were subjected to a migration assay using Boyden chambers in the presence of increasing S100B doses. (**i**) IgG- and anti-S100B-treated injured muscles excised at d7 p.i. and subjected to collagen IV detection by immunohistochemistry and western blotting. (**j**) Macrophages were isolated from IgG- and anti-S100B-treated injured muscles at d3 p.i., cultured for 24 h in the absence or presence of 200 ng S100B/ml, and analyzed by real-time PCR. (**k**) Macrophages were treated with IFN-γ, IL-10 or IL-4 in the absence or presence of the NF-κB inhibitor, Bay11-7085, and analyzed by real-time PCR for *S100b* levels. (**l**) Western blot analysis of S100B in conditioned media of IFN-γ-, IL-10- or IL-4-stimulated peritoneal macrophages. (**m**) Proliferation assay of C2C12 myoblasts cultured in the presence of IgG- or anti-S100B-treated conditioned media from vehicle- or IFN-γ-stimulated peritoneal macrophages (left) and differentiation assay of C2C12 myoblasts cultured in the presence of IgG- or anti-S100B-treated conditioned media from vehicle- or IL-10-stimulated peritoneal macrophages (right). Immunoblots of α-tubulin are included as loading controls in western blots in **e**,**m**. Full-length blots are presented in Supplementary Fig. S7 “Fig. 2”. Results are means ± SEM (n = 6). * *p* < 0.05, **p < 0.01, ***p < 0.001 vs. control. ^#^ *p* < 0.01, ^##^ *p* < 0.001 (d7 vs. d3 p.i., b and c; anti-S100B antibody- vs. IgG-treated, j; BAY11-7085-treated vs. control, k; IFN-γ-treated or IL-10-treated vs. control, m). The scale bar in i represents 50 µm.

Early S100B blockade also reduced infiltration of injured muscle tissue with macrophages as examined at d3 p.i. (Fig. 2a,b and S2a,b). Blocking S100B resulted in smaller amounts of the macrophage marker, MAC3 (Fig. S2d) and numbers of macrophages isolated from muscles (Fig. 2b,c and S2b), and increased levels of the M1 macrophage markers, *Nos2*, *Cd68*, *Ifng*, *Tnfa*, *Il6*, *Cd86*, *Il1b* and *Il12a* and decreased levels of the M2 macrophage markers, *Arg1*, *Cd163a*, *Il4*, *Il10R1*, *Cd206 (Mrc1)* and *Il10* compared to controls (Fig. 2d and S2d). Moreover, higher levels of iNOS and phosphorylated NF-κB, p38 MAPK and ERK1/2, and reduced levels of CD163 and phosphorylated Akt were found in macrophages isolated at d3 p.i. from anti-S100B-treated muscles compared to controls (Fig. 2e). Expression levels of *Dusp1* (MKP-1), a phosphatase that dephosphorylates MAPKs, particularly p38 MAPK, in macrophages and crucially regulates M2 macrophage polarization^29^, were reduced in macrophages isolated from anti-S100B-treated muscles compared to controls (Fig. 2f). Thus, during the first 3 days p.i. S100B might regulate the fine balance of master signaling factors involved in macrophage polarization into M2 phenotype^29,30^. Blocking S100B also caused a reduction of levels of the chemokine receptor, *CCR2*, and *CCl2* ^31^ (Fig. 2f), suggesting that S100B is required for macrophage migration towards damage sites. Indeed, S100B dose-dependently stimulated macrophage migration with maximum effect at 20–200 ng/ml (Fig. 2g). Notably, at d3 and, to a lesser extent, d7 p.i. *Tgfb* levels were higher upon neutralization of S100B (Fig. 2h), and at d7 higher levels of collagen IV were found (Fig. 2i). The higher *Tgfb* levels measured at d3 p.i. after early S100B blockade might reflect M1 macrophages^32^, that represented the dominant macrophage phenotype in anti-S100B-treated muscles (Fig. 2d,e and S2d).

However, at d7 p.i. macrophage numbers were higher in anti-S100B-treated muscles than in controls (Fig. 2b,c and S2b,d,e). This likely reflected the delayed and/or defective reparative process consequent to S100B blockade, exemplified by the persistence of damaged (i.e., IgG-stained) myofibers^33^ (Fig. S2f) and the larger numbers of interstitial myoblasts and myocytes and larger amounts of proliferation and myogenic markers (Fig. 1b–d and S1d,e). At d7 markers of both M1 and M2 macrophages were higher in anti-S100B-treated muscles (Fig. 2d and S2d,e), which pointed to the presence of a mixed M1/M2 population at a time when in control muscles the number of macrophages were low and the vast majority of them were of the M2 phenotype^7,12,29^ (Fig. 2b–d and S2b,d,e). Thus, early after injury S100B is required for timely macrophage infiltration and polarization into the pro-regenerative M2 phenotype, and modulation of collagen deposition.

Treatment with S100B of macrophages isolated at d3 p.i. from control injured muscles reduced expression levels of the proinflammatory markers, *Nos2* and *Cd68*, and increased those of the antiinflammatory markers, *Arg1* and *Cd163a*, these effects being more pronounced in macrophages from anti-S100B-treated muscles (Fig. 2j). S100B also increased *Dusp1* levels (Fig. 2j) in accordance with the reduced *Dusp1* levels detected in macrophages from anti-S100B-treated muscles (see Fig. 2f). At comparable levels to those released early after muscle injury S100B exerted on macrophages similar effects to the antiinflammatory cytokines, IL-4 and IL-10, that is a decrease in *Nos2* and *Cd68* levels and increase in *Arg1* and *Cd163a* levels (Fig. S2g). Thus, early after acute muscle injury released S100B might exert antiinflammatory effects and promote macrophage M2 polarization.

S100B is not expressed in tissue macrophages in normal physiological conditions^15^. However, *S100b* was expressed in macrophages isolated from injured muscles at d3 and d7 p.i. (Fig. S2h) and in macrophages infiltrating injured muscles (Fig. S2i), and IFN-γ, IL-10 and IL-4 upregulated *S100b* albeit with different potencies (Fig. 2k). IFN-γ-, but not IL-4-induced upregulation of *S100b* in macrophages was dependent on NF-κB, whereas inhibition of NF-κB greatly enhanced IL-10-induced *S100b* upregulation (Fig. 2k). Upon activation with IFN-γ, IL-10 or IL-4 macrophages released S100B (Fig. 2l), and pretreatment of CM from IFN-γ-treated (M1) macrophages with S100B neutralizing antibody reduced macrophage CM-induced myoblast proliferation (Fig. 2m, left panel) in line with the known mitogenic activity of S100B^19,20^. Conversely, S100B blockade increased the ability of macrophage CM to induce myogenin (Fig. 2m, right panel) in line with the known anti-myogenic activity of S100B^18,20^, however blockade of S100B in CM from IL-10-treated (M2) macrophages was without effects on CM-induced increase in myogenin levels (Fig. 2m, right panel) possibly because myoblast differentiation was maximally stimulated under the present experimental conditions. Thus, macrophage-derived S100B might participate in muscle regeneration along with myofiber-released S100B by virtue of its ability to promote M1-M2 macrophage transition and its mitogenic and anti-myogenic effects towards myoblasts.

### S100B regulates muscle regeneration by acting on both myoblasts and macrophages

Treatment with clodronate, a drug that kills monocytes^34^, robustly reduced the number of interstitial cells (Fig. S3a,b), the extent of macrophage infiltration (Fig. S3c) and MAC3 levels (Fig. S3d) in the presence of degenerating (i.e. IgG-stained) myofibers (Fig. S3e) at d3 p.i., as expected^35^. Blocking S100B in clodronate-treated muscles resulted in even smaller numbers of interstitial cells (Fig. S3b), no effects on macrophage numbers (Fig. S3c), and a further decrease in numbers of PAX7^+^, MyoD^+^, myogenin^+^ and Ki67 cells^+^ (Fig. S3f,g). The few macrophages detected in clodronate-treated muscles were of the M1 phenotype given their extremely low CD163a levels and high iNOS levels, and S100B blockade increased iNOS levels (Fig. S3d).

### S100B is likewise required during the M2 macrophage phase for efficient regeneration

**Figure 3:**
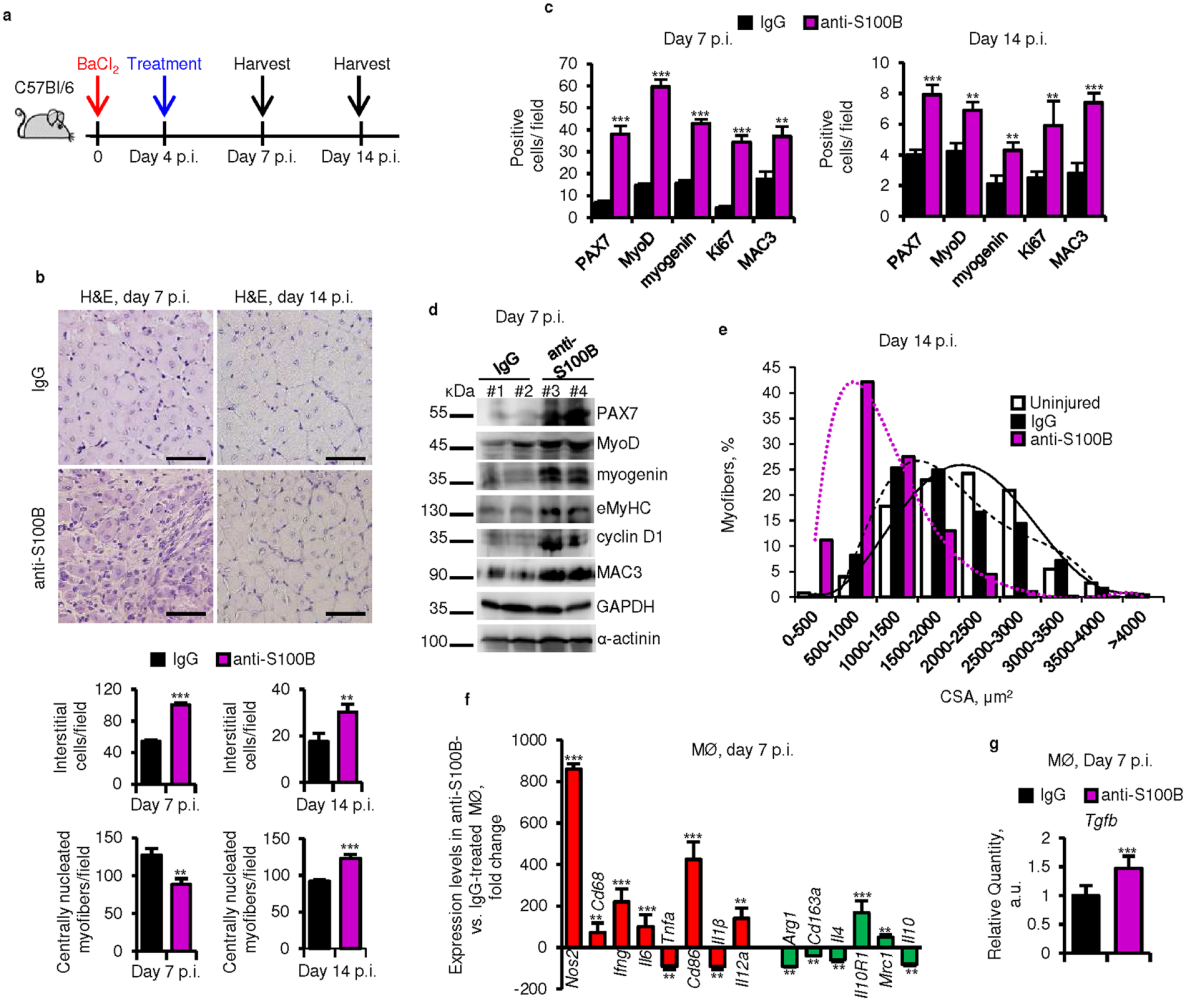
S100B is required during the macrophage M2 phase for efficient regeneration. (**a**) Injured TA muscles were injected with IgG or anti-S100B antibody at d4 p.i. Treated muscle were excised at d7 or d14 p.i. (**b**) Histology of muscle tissue (upper panel) and counts of interstitial cells and centrally nucleated myofibers (lower panel). (**c**) PAX7^+^, MyoD^+^, myogenin^+^, MAC3^+^ and Ki67^+^ cell counts (also see Fig. S4b). (**d**) Western blots of the indicated proteins in homogenates of IgG- and anti-S100B-treated muscles. Immunoblots of GAPDH and α-actinin are included as loading controls. (**e**) Myofiber size distribution at d14 p.i. of uninjured muscles and IgG- and anti-S100B-treated injured muscles. (**f,g**) Macrophages isolated from IgG- and anti-S100B-treated injured muscles and analyzed by real-time PCR. Full-length blots are presented in Supplementary Fig. S7 “Fig. 3”. Results are means ± SEM (n = 6). **p < 0.01, ***p < 0.001 vs. control. The scale bar in (**b**,**c** and **f**) represents 50 μm.

When anti-S100B antibody was administered to injured muscles at d4 p.i., i.e. around the peak of the M2 macrophage phase^7,29^ (Fig. 3a), muscle regeneration was likewise delayed. As analyzed at d7, blocking S100B caused a significant increase in the number of proliferating cells, myoblasts, myocytes and macrophages and levels of PAX7, MyoD, myogenin, cyclin D1, eMyHC and MAC3, and a decrease in the number of centrally nucleated myofibers (Fig. 3b–d and S4a,b). At d7 myofibers displayed IgG staining indicative of defective repair whereas no such staining was detected in control muscles (Fig. S4c). Blocking S100B at d4 p.i. also resulted at d14 in higher numbers of interstitial PAX7^+^, MyoD^+^, myogenin^+^, Ki67^+^, and MAC3^+^ cells and centrally nucleated myofibers, and decreased mean cross-sectional area compared to controls (Fig. 3b,c,e), indicative of delayed regeneration. Compared to controls, in anti-S100B-treated muscles macrophages isolated at d7 prevalently were of the M1 phenotype given their higher levels of *Nos2*, *Cd68*, *Ifng*, *Il6*, *Cd86*, and *Il12a* and smaller levels of *Arg1*, *Cd163a*, *Il4* and *IL10* (Fig. 3f) and expressed larger levels of *Tgfb* (Fig. 3g). S100B blockade also resulted in greater collagen deposition (Fig. S4c). The higher number of proliferating myoblasts and increased expression levels of proinflammatory markers at d7 p.i. in anti-S100B-treated muscles (Fig. 3c,d,f) suggested that at d4–d6 also S100B might promote M1/M2 macrophage transition and likely the pro-regenerative activity of M2 macrophages; myofibers so regenerated would stop releasing (or at least reduce the release of) factors with ability to support sterile inflammation, to prolong the myoblast proliferation phase and to delay regeneration. Incidentally, the potential decrease in the myoblast number following late blockade of the mitogenic S100B might have been obscured by the release of mitogenic factors by M1 macrophages which were numerous (Fig. 3d,f) and represented the dominant macrophage population in the injured muscles that received the anti-S100B antibody at d4 (Fig. 3f).

### S100B’s ability to promote regeneration of acutely injured muscles requires RAGE at early, but not mid-late regeneration phases

Regeneration is significantly delayed in acutely injured *Ager* ^−/−^ muscles pointing to a promyogenic and pro-regenerative role of RAGE^9^. *Ager* deletion results in increased asymmetric division of myoblasts and delayed albeit enhanced infiltration of injured muscles with macrophages upon acute injury^9^. Control *Ager* ^−/−^ macrophages differed from wild-type macrophages as examined at d7 p.i., i.e. shortly beyond the peak of macrophage infiltration of *Ager* ^−/−^ muscles. We found higher levels of M1 macrophage (e.g. *Nos2*, *Cd68*, *Ifng*, *Il6*, *Tnfa*, *Cd86*, *Il1b*, and *Il12a*) markers and lower levels of M2 macrophage (e.g. *Arg1*, *Cd163a*, *Il4*, and *Il10*) markers (yet with higher levels of *Il10R1*, *Mrc1* and *Tgfb*) in injured *Ager* ^−/−^ muscles than in wild-type muscles (Fig. S5a). These results (i) suggested that RAGE signaling is required for correct timing of macrophage polarization into M2 phenotype, and (ii) contributed to explain the prolonged proliferation phase of injured *Ager* ^−/−^ muscles^9^ likely due to prolonged release of mitogenic factors by M1 macrophages.

When anti-S100B antibody was administered at d1 p.i. to *Ager* ^−/−^ muscles (Fig. S5b) no effects could be documented at d3 and d5 in terms of numbers of interstitial PAX7^+^, MyoD^+^, myogenin^+^, MAC3^+^ and Ki67^+^ cells, and centrally nucleated myofibers (Fig. S5c,d) and expression levels of either M1 or M2 markers compared to *Ager* ^−/−^ controls (Fig. S5e). Also, S100B’s stimulatory effect on macrophage migration proved RAGE-dependent as it was robustly attenuated in macrophages from *Ager* ^−/−^ mice (Fig. S5f). Thus, during the first few days after acute injury S100B stimulates myoblast proliferation, attracts macrophages and promotes macrophage M2 polarization in a RAGE-dependent manner.

**Figure 4:**
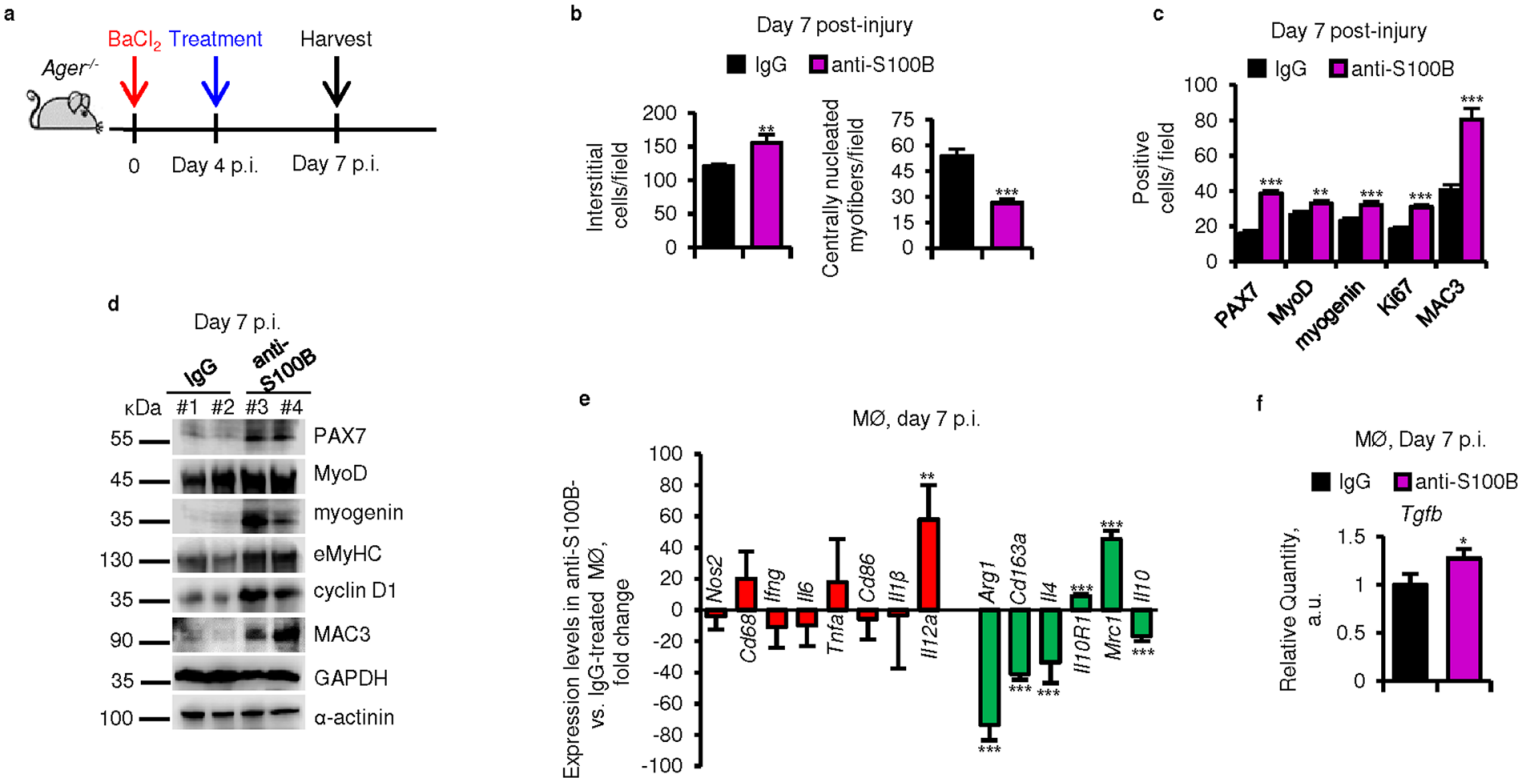
S100B’s ability to promote regeneration of acutely injured skeletal muscles requires RAGE at early, but not mid-late regeneration phase. (**a**) Injured *Ager* ^−/−^ TA muscles were injected with IgG or anti-S100B antibody at d4 p.i. and excised at d7 p.i. (**b**) Counts of interstitial cells and centrally nucleated myofibers (also see Fig. S5h). (**c**) PAX7^+^, MyoD^+^, myogenin^+^, MAC3^+^ and Ki67^+^ cell counts (also see Fig. S5i). (**d**) Western blots of the indicated proteins in homogenates of IgG- and anti-S100B-treated *Ager* ^–/–^ muscles. Immunoblots of GAPDH and α-actinin are included as loading controls. Full-length blots are presented in Supplementary Fig. S8 “Fig. 4”. (**e**,**f**) Macrophages isolated at d7 p.i. from injured *Ager* ^−/−^ muscles and analyzed by real-time PCR to measure the indicated macrophage markers (**e**) and *Tgfb* (**f**). Results are means ± SEM (n = 6). *p < 0.05, **p < 0.01, ***p < 0.001 vs. control.

However, when anti-S100B antibody was administered at d4 p.i. to *Ager* ^−/−^ muscles and muscles were analyzed at d7 (Fig. 4a), the degeneration and myoblast proliferation phases were prolonged, as indicated by the higher number of interstitial PAX7^+^, MyoD^+^ and myogenin^+^ cells and macrophages, the higher levels of proliferation and myogenic differentiation markers and MAC3 and the smaller number of centrally nucleated myofibers compared to *Ager* ^−/−^ controls (Fig. 4b–d and S5h,i). Notably, no significant differences could be found between IgG- and anti-S100B-treated *Ager* ^−/−^ muscles in terms of expression levels of M1 macrophage markers (excepting increased *Il12a levels*) (Fig. 4e) as opposed to the increased expression levels of M1 markers detected in anti-S100B-treated wild-type macrophages (compare Fig. 4e with Fig. 3f). This suggested that RAGE was required for S100B to mitigate the inflammatory response late after injury in wild-type muscles. Contrariwise, compared to *Ager* ^−/−^ controls blocking S100B at d4 caused similar changes in expression levels of M2 markers, *Tgfb* levels and collagen deposition to those observed in wild-type muscles (compare Fig. 4e,f and S5h with Fig. 3f,g). These results suggested that during the M2 macrophage phase S100B might activate a receptor other than RAGE (in injured *Ager* ^−/−^ muscles) or in addition to RAGE (in injured wild-type muscles) to promote M2 macrophage polarization and, hence, muscle regeneration.

### Late blockade of S100B results in altered FGFR1 signaling

**Figure 5:**
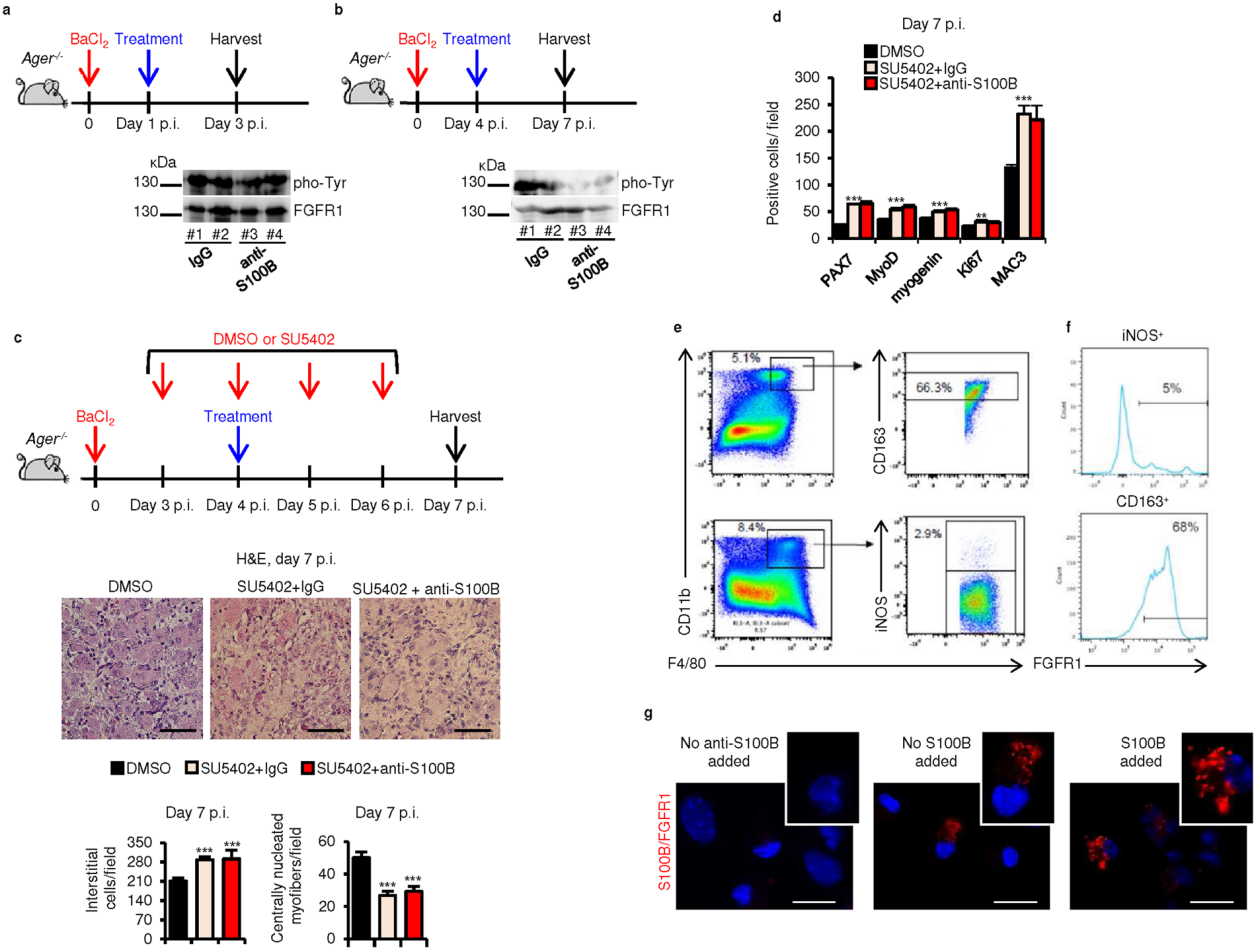
Late blockade of S100B results in altered bFGF/FGFR1 signaling. (**a**,**b**) Injured *Ager* ^−/−^ TA muscles were injected with IgG or anti-S100B antibody at d1 (**a**) or d4 (**b**) p.i. and excised at d3 or d7, respectively. Muscle homogenates were subjected to western blotting for detection of total and phosphorylated (pho-Tyr)-FGFR1. ^#^Denotes experiment number. Full-length blots are presented in Supplementary Fig. S8 “Fig. 5”. (**c**) Conditions were as in (**b**) except that mice were treated with vehicle or SU5402 at d3, d4, d5 and d6 p.i. Muscles were excised at d7 p.i. and analyzed by histology for counts of interstitial cells and centrally nucleated myofibers. (**d**) PAX7^+^, MyoD^+^, myogenin^+^, MAC3^+^ and Ki67^+^ cell counts (also see Fig. S6b). (**e**) Flow cytometry analysis of CD163 and iNOS on CD11b^+^-F4/80^+^ cells isolated at d4 p.i. from *Ager* ^–/–^ muscles. Numbers above the gates indicate the frequency of positive cells. (**f**) Flow cytometry analysis of FGFR1 on CD11b^+^-F4/80^+^-CD163^+^ and CD11b^+^-F4/80^+^-iNOS^+^ cells isolated at d4 p.i. from *Ager* ^−/−^ muscles. Numbers above the gates indicate the frequency of positive cells. (**g**) Macrophages isolated at d4 p.i. from injured *Ager* ^−/−^ muscles and analyzed by proximity ligation assay for detection of S100B-bFGF-FGFR1 complexes. Results are means ± SEM (n = 6). **p < 0.01, ***p < 0.001 vs. control. The scale bar represents 50 µm in (**c**) and 100 µm in (**g**).

As in high-density myoblast cultures S100B enhances bFGF/FGFR1 signaling and simultaneously blocks RAGE^20^ and activates the bFGF-FGFR1 complex in *Ager* ^−/−^ myoblasts irrespective of their density^21^, we examined the possibility that S100B might activate FGFR1 in acutely injured *Ager* ^−/−^ muscles. Blocking S100B at d1 p.i. caused no changes in FGFR1 phosphorylation (activation) levels as measured at d3 (Fig. 5a **)** in agreement with the RAGE-dependence of S100B’s effects early after injury. However, blocking S100B at d4 caused reduced FGFR1 phosphorylation levels as measured at d7 (Fig. 5b), pointing to a requirement of S100B for optimal FGFR1 signaling late after injury. Moreover, muscles of injured *Ager* ^−/−^ mice that had been treated at d3–d6 p.i. with SU5402, a specific antagonist to FGFR1 tyrosine kinase, showed at d7 larger numbers of interstitial PAX7^+^, MyoD^+^, myogenin^+^, Ki67^+^, and MAC3^+^ cells and smaller numbers of regenerating myofibers compared to vehicle-treated controls (Fig. 5c,d and S6a,b), indicative of delayed regeneration. However, as examined at d7 p.i. blocking S100B at d4 p.i. in SU5402-treated *Ager* ^−/−^ muscles caused no significant changes in the (immuno)histological picture and numbers of interstitial cells (Fig. 5c,d and S6b) and expression levels of M1 and M2 macrophage markers (excepting higher *Nos2* levels) compared to IgG-injected controls (Fig. S6c). Thus, functional FGFR1 was required late after injury for S100B to promote muscle tissue repair in *Ager* ^−/−^ muscles.

While S100B activates bFGF/FGFR1 in *Ager* ^−/−^ myoblasts thereby stimulating their proliferation^20^, we examined whether S100B interacts with FGFR1 in *Ager* ^−/−^ macrophages. As analyzed at d4 p.i., control injured *Ager* ^−/−^ muscles exhibited ~3% of CD11b^+^-F4/80^+^-iNOS^+^ (M1) macrophages and ~66% of CD11b^+^-F4/80^+^-CD163^+^ (M2) macrophages, and FGFR1 was expressed in ~72% of CD11b^+^-F4/80^+^-CD163^+^ (M2) macrophages and ~5% of CD11b^+^-F4/80^+^-iNOS^+^ (M1) macrophages (Fig. 5e,f). Thus, at d4 p.i. FGFR1 was prevalently, if not exclusively expressed in M2 macrophages in injured *Ager* ^−/−^ muscles. As investigated by proximity ligation assay S100B associated with FGFR1 in isolated M2 *Ager* ^−/−^ macrophages (Fig. 5g), and FGFR1 co-immunoprecipitated with S100B following treatment of isolated M2 *Ager* ^−/−^ macrophages with S100B (Fig. S6d). Collectively, these results suggested that in *Ager* ^−/−^ muscles S100B might activate the bFGF/FGFR1 complex to expand the myoblast population and promote M2 macrophages’ pro-regenerative activity during the mid-late phase of the regeneration process.

### Persistence and/or accumulation of S100B at damage sites prolongs the M1 macrophage inflammatory phase and dampens muscle regeneration

**Figure 6:**
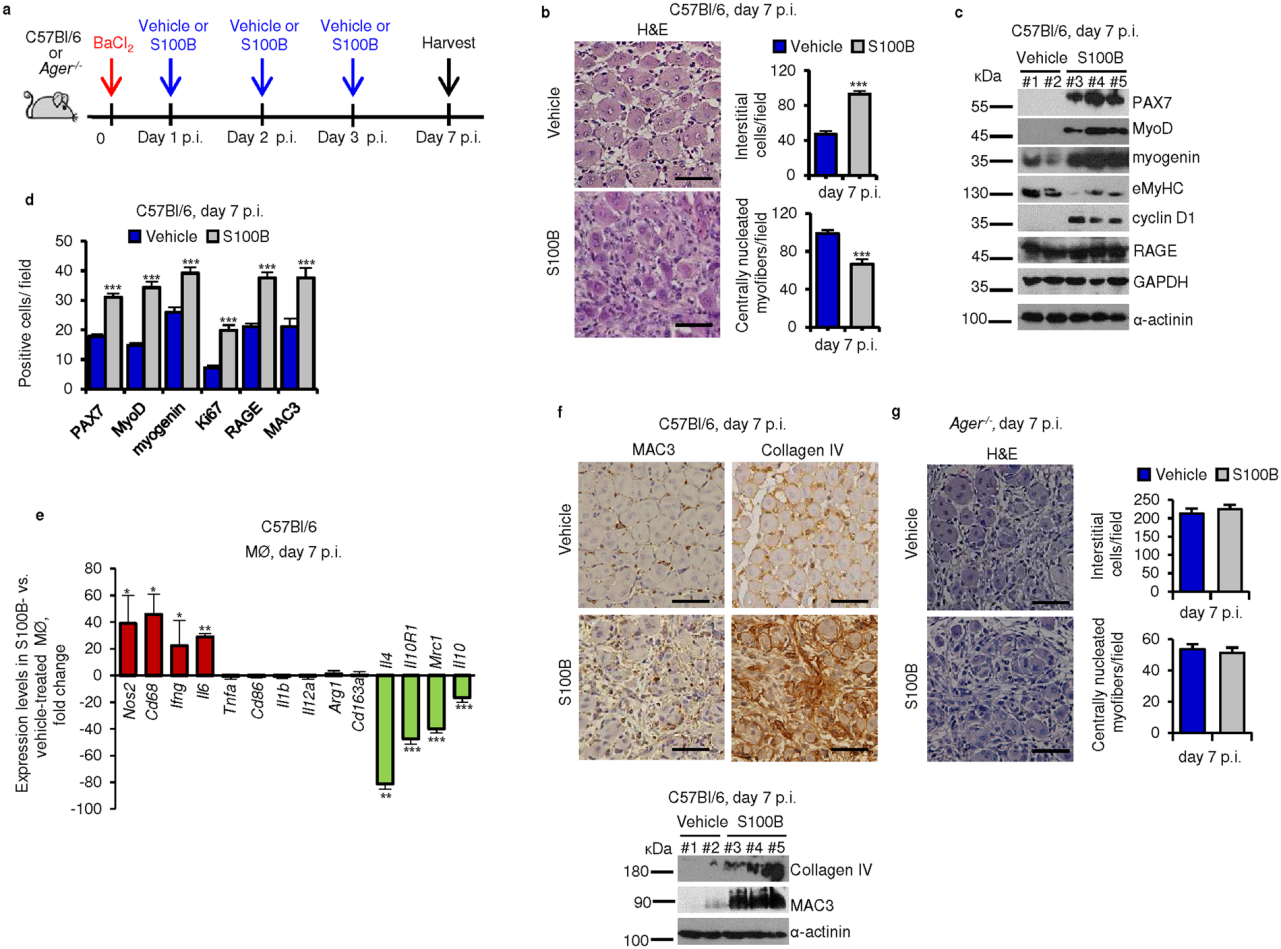
Persistence of S100B at damage sites following acute muscle injury prolongs the M1 macrophage (inflammatory) phase and dampens muscle regeneration. (**a**) Injured wild-type or *Ager* ^−/−^ TA muscles were injected with vehicle or S100B at d1, d3 and d5 p.i., excised at d7 p.i. and analyzed as detailed in b-f for wild-type TA and in g for *Ager* ^−/−^ TA. (**b**) Histology and counts of interstitial cell and centrally nucleated myofiber numbers. (**c**) Muscle homogenates were subjected to western blotting for detection of the indicated proteins. Immunoblots of GAPDH and α-actinin are included as loading controls. Full-length blots are presented in Supplementary Fig. S8 “Fig. 6”. (**d**) Counts of cell types based on immunohistochemistry for the indicated antigens. (**e**) Macrophages were isolated from muscles and subjected to real-time PCR for determination of levels of the indicated genes. (**f**) Muscles were analyzed for MAC3 or collagen IV expression by immunohistochemistry and western blotting. Immunoblots of α-actinin are included as loading controls. Full-length blots are presented in Supplementary Fig. S8 “Fig. 6”. (**g**) Injured *Ager* ^−/−^ TA muscles were injected with S100B as described in (**a**) and analyzed as described in (**b**). Results are means ± SEM (n = 6). **p < 0.01, ***p < 0.001 vs. control. The scale bar represents 50 µm in (**b**,**f** and **g**).

We analyzed the impact on regeneration of persistence of relatively high levels of S100B in injured wild-type muscles during the first few days after injury. Intramuscular (i.m.) injection with S100B (60 ng/muscle) for three consecutive days after acute injury of wild-type muscles (Fig. 6a) caused a remarkable delay of regeneration as evidenced by the greater number of interstitial PAX7^+^, MyoD^+^, myogenin^+^, proliferating (Ki67^+^) and RAGE^+^ cells (Fig. 6b–d), and reduced number of centrally nucleated myofibers (Fig. 6b); greater macrophage infiltration (Fig. 6d,f); reduced M1/M2 macrophage transition (Fig. 6e); and, enhanced collagen IV deposition (Fig. 6f), compared to vehicle injected controls, as analyzed at d7 p.i. These S100B’s effects were RAGE-dependent as no effects of i.m. injection with S100B could be documented in *Ager* ^−/−^ muscles (Fig. 6g). Thus, a rapid clearance of released S100B following acute injury is required for timely resolution of inflammation and muscle regeneration.

### High levels of released S100B in *mdx* muscles prolong the M1 macrophage inflammatory phase and dampen muscle regeneration

**Figure 7:**
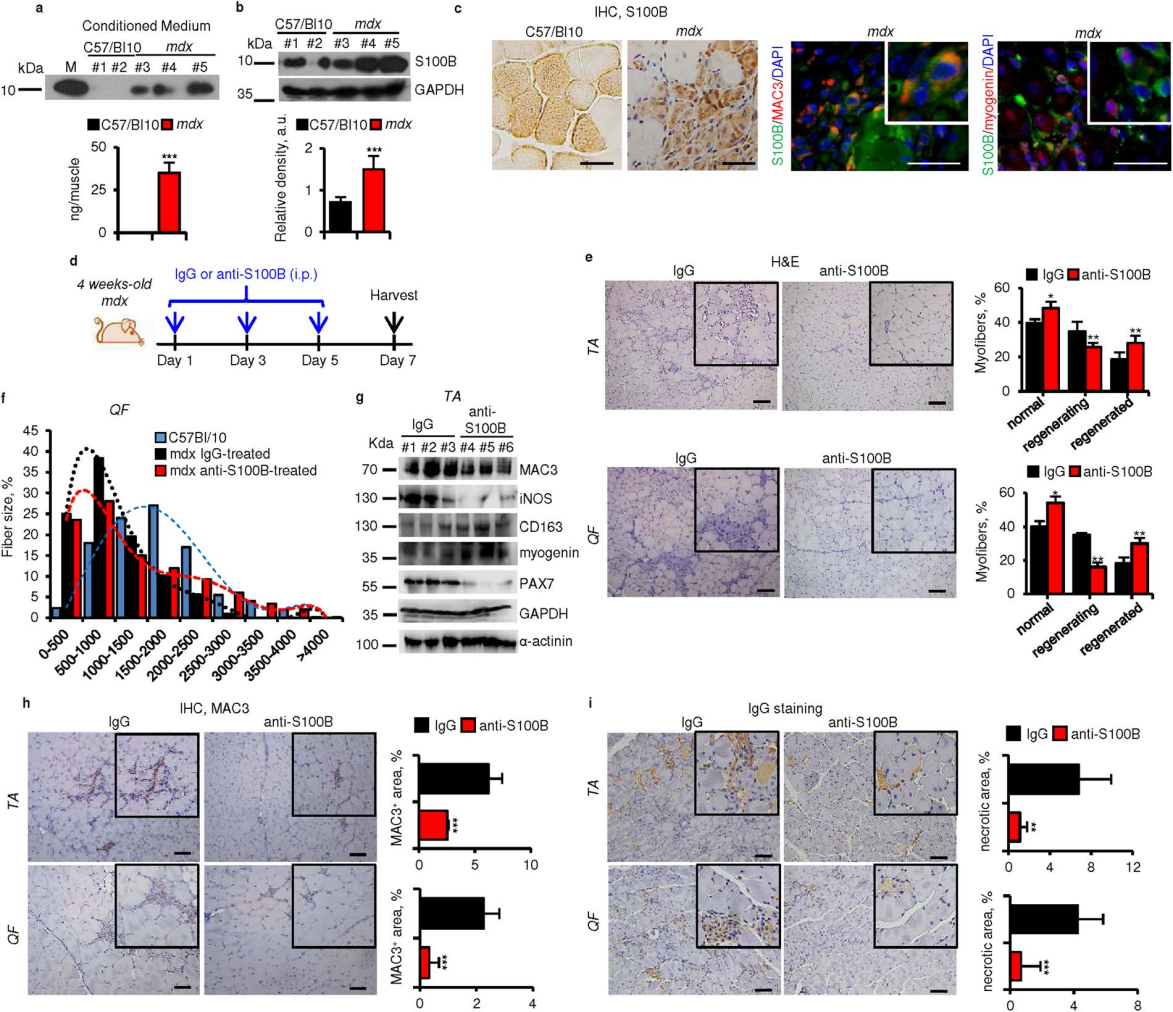
High levels of released S100B in *mdx* muscles prolong the M1 macrophage inflammatory phase and dampen muscle regeneration. (**a**,**b**) Uninjured, untreated TA muscles of 4-wk-old wild-type and *mdx* mice were excised and incubated in PBS for 2 h at 4 °C. Conditioned media were collected, TCA precipitated and subjected to western blotting for detection of released S100B. M denotes purified S100B (10 ng). ^#^Denotes experiment number. Immunoblots of GAPDH and α-actinin are included as loading controls. Full-length blots are presented in Supplementary Fig. S8 “Fig. 7”. (**c**) Conditions were as in (**a**,**b**) excepting that muscles were fixed and paraffin-included. Cross-sections were subjected to immunohistochemistry (left panels) for detection of S100B. *Mdx* TA muscles processed as above were subjected to double immunofluorescence for detection of S100B (green) and either MAC3 (red) or myogenin (red) (right panels). (**d**–**i**) Four-wk-old *mdx* mice were i.p. injected with either IgG or anti-S100B antibody (1 µg/mouse) for 3 days every other day and sacrificed two days after the last injection (**d**). TA and QF muscles were subjected to histology for counts of normal, regenerating and regenerated myofibers (**e**), QF muscles were analyzed for myofiber size distribution (**f**), TA muscle homogenates were subjected to western blotting for detection of the indicated proteins (**g**), TA and QF muscles were subjected to either MAC3 immunohistochemistry for measurement of MAC3^+^ areas (**h**) or to IgG staining for measurement of necrotic areas (**i**). Immunoblots of GAPDH and α-actinin are included as loading controls. Full-length blots are presented in Supplementary Fig. S8 “Fig. 7”. Results are means ± SEM (n = 6) (**e**,**h**,**i**). ^#^Denotes experiment number (**g**). **p < 0.01, ***p < 0.001 vs. control. The scale bar represents 50 µm in (**c,e,h,i**).

Next, we examined S100B in muscles of *mdx* mice, an animal model of muscular dystrophy, characterized by a high inflammatory component. Levels of S100B release were several times larger from TA *mdx* muscles (~30 ng/ml) than from wild-type controls (essentially no release) (Fig. 7a), and TA *mdx* muscle homogenates contained 2 times more S100B than did wild-type controls (Fig. 7b) likely consequent to shedding of intramyofiber S100B and a high number of S100B^+^ interstitial cells (Fig. 7c) which were macrophages and cells of the myogenic lineage in part (Fig. 7c). I.p. injection of *mdx* mice with anti-S100B antibody (1 µg/mouse, every other day for a total of three injections) (Fig. 7d) caused a decrease in the number of regenerating myofibers and increase in the number of normal and regenerated myofibers in TA and *Quadriceps femoralis* (QF) (Fig. 7e), an increase in the number of normally sized and large myofibers (Fig. 7f), a robust reduction of macrophage infiltration of TA and QF (Fig. 7g,h), and a reduction of the number of necrotic myofibers (Fig. 7i), compared to IgG-injected controls, as examined 2 days after treatment. These changes were paralleled by decreased levels of MAC3, the M1 macrophage marker iNOS and the myoblast proliferation marker PAX7 and increased levels of the M2 macrophage marker CD163 and myocyte marker myogenin in TA muscles from anti-S100B-treated vs. IgG-treated *mdx* mice (Fig. 7g). These findings suggested that accumulation of extracellular S100B in *mdx* muscles contributed to the histopathology of muscular dystrophy, and S100B neutralization significantly improved histology.

## Discussion

We report the unprecedented observation that S100B, released from damaged muscles early after acute injury, is required for correct timing of muscle regeneration by virtue of its action on myoblasts and macrophages, however its persistence at relatively high levels dampens regeneration. Early after acute injury released S100B expands the myoblast population, stimulates myoblast migration, recruits macrophages to damage sites, and promotes polarization of infiltrating macrophages from the M1 (proinflammatory) phenotype to the M2 (antiinflammatory and pro-regenerative) phenotype. Early neutralization of released S100B results in reduced numbers of proliferating and differentiating myoblasts and of macrophages as investigated at d3, along with reduced numbers of regenerating myofibers at d7 and increased numbers of thin, regenerating myofibers at d14, compared to controls. The reduced infiltration with M1 macrophages consequent to S100B blockade likely causes a reduction of phagocytosis of cell debris and, hence, a prolongation of the inflammatory phase as indicated by the increased levels of proinflammatory factors and decreased levels of antiinflammatory factors in macrophages. This suggests that, besides acting on myoblasts to stimulate their proliferation and migration, early after injury released S100B might promote macrophage polarization into the pro-regenerative M2 phenotype during the next few days. Although not addressed here, the reduced levels of *Il4* caused by S100B blockade might negatively impact fibro/adipogenic progenitors shown to be required for rapid clearance of necrotic debris and timely and complete regeneration of muscle tissue^4,5^. Likewise, the reduced levels of *Il10* caused by early S100B blockade might negatively impact the pro-regenerative activity of regulatory T cells^36^. Notably, although the fewer macrophages detected in anti-S100B-treated muscles are of the M1 phenotype and thus expected to release mitogenic factors towards myoblasts including S100B itself, they cannot compensate for the absence/reduction of S100B level likely because of their low abundance. A role of S100B as a regulator of both myoblasts and infiltrating macrophages is supported by the finding that S100B stimulates myoblast proliferation in clodronate-treated injured mice. Interestingly, as investigated at d7 p.i., early or mid-late S100B blockade results in high levels of collagen deposition at damage sites, in accordance with the elevated levels of *Tgfb* measured at d3 and d7 in macrophages isolated from anti-S100B-treated muscles. By regulating inflammatory cell activity, especially *Tgfb* expression, S100B affects the deposition of collagen that acts as a scaffold for the newly formed myofibers.

We also report the unprecedented observation that S100B is transiently induced in and secreted by activated macrophages. As maximal infiltration of acutely injured muscle tissue with M1 macrophages occurs around d3 p.i.^8,29^, it is possible that early after injury S100B expands the myoblast population both directly by acting on myoblasts and indirectly by attracting M1 macrophages to damage sites and enhancing their mitogenic activity towards myoblasts. On the other hand, since in acutely injured muscles M2 macrophages reach their maximum around d4 p.i.^8,29^, myofiber- and/or macrophage-derived S100B might promote M2 polarization of macrophages and their pro-regenerative capacity at d4–d7 p.i.

**Figure 8:**
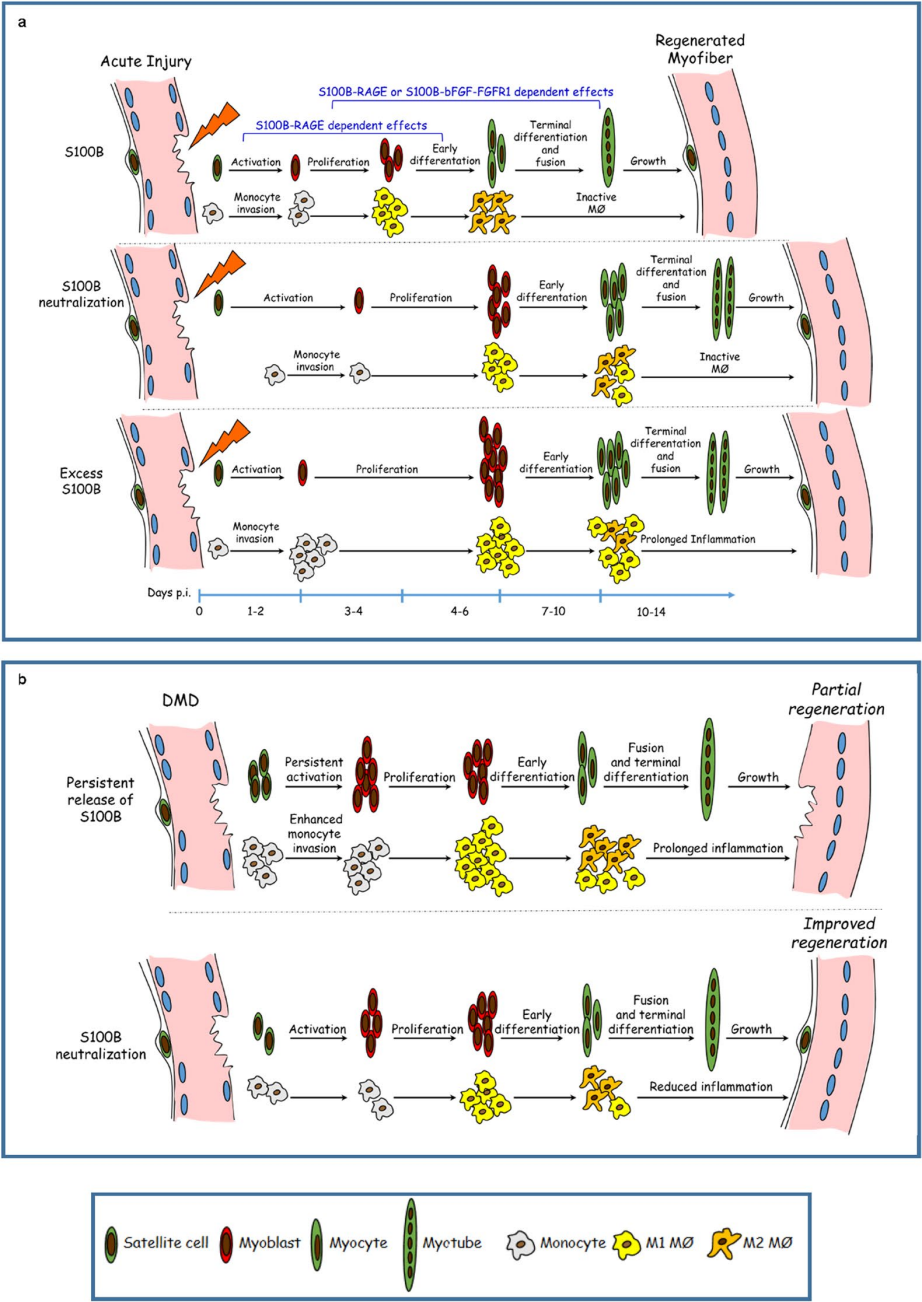
Schematic of the proposed role of S100B in muscle regeneration following acute injury and in *mdx* muscles. (**a**) Top panel. S100B, released from damaged myofibers and infiltrating macrophages, attracts macrophages to damage sites, promotes M1/M2 macrophage switch and stimulates myoblast proliferation RAGE-dependently during the first 3–4 days p.i. Depending on local conditions (e.g., myoblast density, S100B levels and bFGF availability) S100B can stimulate myoblast proliferation and M1/M2 macrophage switch either by engaging RAGE or by enhancing bFGF-FGFR1 signaling in this time interval and thereafter. Effects of S100B on attraction of macrophages to damage sites are strictly RAGE-dependent. Middle panel. Blocking S100B early after injury delays macrophage infiltration and M1/M2 macrophage switch with resultant delayed regeneration. Bottom panel. Defective clearance or excess release of S100B resulting in persistence of S100B at damage sites prolongs the M1 (proinflammatory) macrophage phase via RAGE engagement with resultant delayed regeneration. (**b**) In muscular dystrophy (DMD) levels of released S100B (from damaged myofibers and infiltrating macrophages) are high, which might contribute to a state of unrestricted inflammation and resultant defective regeneration (top panel). Indeed, blocking S100B in this condition reduces macrophage infiltration and inflammation and improves muscle regeneration (bottom panel).

S100B’s effects on myoblasts and macrophages following acute muscle injury are strictly dependent on its canonical receptor, RAGE, during the first 3 days p.i. (Fig. 8a); early neutralization of S100B in *Ager* ^−/−^ muscles results in a similar, if not identical regeneration pattern to *Ager* ^−/−^controls. Contrarily, blocking S100B at 4d p.i. in wild-type muscles determines delayed regeneration, causing a prolongation of the inflammatory phase and likely, macrophage-dependent myoblast proliferation at the expense of myofiber repair, and effects of late S100B blockade are similar in wild-type and *Ager* ^−/−^ mice. The present results suggest that S100B’s effects at d4–d7 p.i. might rely on activation of either RAGE or the bFGF-FGRF1 complex depending on local conditions (i.e., myoblast density, local S100B and bFGF concentrations) in wild-type muscles and on bFGF-FGRF1 complex in *Ager* ^−/−^ muscles (Fig. 8a). Indeed, blocking S100B at d4 p.i. in *Ager* ^−/−^ muscles reduces FGFR1 activity and is without effects in *Ager* ^−/−^ mice in which FGFR1 signaling has been pharmacologically blocked. Our results also suggest that at d4–d7 p.i. S100B can interact with FGFR1 in M2 macrophages likely stimulating their pro-regenerative activity. Given the proangiogenic properties of bFGF^37^, we cannot exclude that S100B might interact with bFGF/FGFR1 to stimulate angiogenesis, another means to promote muscle repair.

Our results suggest that S100B is an important molecular determinant of muscle regeneration after acute injury by virtue of its regulatory effects on myoblasts and macrophages. However, released S100B must be rapidly cleared, as observed following acute muscle injury^21^, to permit timely muscle damage resolution and efficient regeneration. Indeed, high S100B levels at damage sites as obtained by repeated S100B injections into acutely injured muscles prolong the recruitment of inflammatory cells, the M1 macrophage phase and myoblast proliferation at the expense of differentiation thus delaying muscle regeneration, which recalls the histopathology found in *mdx* muscles during the acute phase of the pathology (Fig. 8b). Also, high extracellular S100B levels result in fibrosis, which might have important consequences in those muscle pathologies associated with chronic tissue damage. Indeed, S100B is released at relatively high levels from 4-weeks old *mdx* muscles, which are characterized by necrosis, elevated macrophage infiltration and compromised regeneration^38^. In *mdx* muscles S100B contributes to macrophage-mediated inflammation and defective regeneration; blocking S100B improves histopathology by reducing macrophage recruitment and muscle degeneration, and promoting regeneration (Fig. 8b). Thus, local levels of extracellular S100B dictate its beneficial or detrimental effects on muscle regeneration following acute muscle injury or in DMD. Based on our results, S100B might be regarded as a potential molecular target in DMD.

## Electronic supplementary material

Supplementary Information
