# Supplementary material for: Levels of S100B protein drive the reparative process in acute muscle injury and muscular dystrophy
Source: Sci Rep. 2017 Oct 2;7:12537. doi: 10.1038/s41598-017-12880-9 (PMC5624904; doi:10.1038/s41598-017-12880-9)
Supplement: Supplementary file 1 — Supplementary Information [file 41598_2017_12880_MOESM1_ESM.pdf]

Supplementary Material

Levels of S100B protein drive the reparative process in acute muscle injury and muscular dystrophy

Francesca Riuzzi[1,4]*, Sara Beccafico[1,4]*, Roberta Sagheddu[1,4], Sara Chiappalupi[1,4], Ileana Giambanco[1], Oxana Bereshchenko[2], Carlo Riccardi[2], Guglielmo Sorci[1,4]** and Rosario Donato[1,3,4]**

[1]Department of Experimental Medicine, [2]Department of Medicine, [3]Centro Universitario per la Ricerca sulla Genomica Funzionale, [4]Istituto Interuniversitario di Miologia, Perugia Medical School, University of Perugia, Piazza Lucio Severi 1, 06132 Perugia, Italy[2]

Supplementary Materials and Methods

Supplementary Table 1

Primary antibodies used in immunohistochemistry and immunofluorescence			
**Antibodies**	**Host animal**	**Dilution**	**Source**
PAX7	Mouse	1:50	R&D Systems
MyoD clone 5.8A	Mouse	1:50	Santa Cruz Biotechnology
Myogenin	Mouse	1:50	Santa Cruz Biotechnology
RAGE	Goat	1:50	Santa Cruz Biotechnology
Ki67	Mouse	1:200	Cell Signaling Technology
Collagen IV	Rabbit	1:400	Novus Biologicals
MAC3	Rat	1:50	BD Biosciences
CD163	Rabbit	1:200	Bioss
NOS2	Rabbit	1:100	Santa Cruz Biotechnology
S100B	Mouse	1:100	BD Biosciences
FGFR1	Mouse	1:100	Chemicon

Secondary antibodies used in immunofluorescence				
**Host**	**Against**	**Fluorophore**	**Dilution**	**Source**
Donkey	Mouse IgG	Alexa Fluor 488	1:200	Invitrogen
Donkey	Rabbit IgG	Alexa Fluor 488	1:200	Invitrogen
Donkey	Rabbit IgG	Alexa Fluor 594	1:200	Invitrogen
Donkey	Rat IgG	Alexa Fluor 594	1:200	Invitrogen

Supplementary Table 2

**Primary antibodies used in Western blotting**			
**Antibodies**	**Host animal**	**Dilution**	**Source**
PAX7	Mouse	1:500	R&D Systems
MyoD clone 5.8A	Mouse	1:1000	Santa Cruz Biotechnology
Myogenin	Mouse	1:1000	Santa Cruz Biotechnology
RAGE	Goat	1:1000	Santa Cruz Biotechnology
eMyHC	Mouse	1:500	Monosan
Ciclin D1	Rabbit	1:1000	Santa Cruz Biotechnology
Collagen IV	Rabbit	1:2000	Novus Biologicals
MAC3	Rat	1:1000	BD Biosciences
CD163	Rabbit	1:1000	Bioss
NOS2	Rabbit	1:1000	Santa Cruz Biotechnology
S100B	Mouse	1:5000	BD Biosciences
FGFR1	Mouse	1:1000	Chemicon
phosphorylated-Tyr clone PY20	Mouse	1:1000	Santa Cruz Biotechnology
phosphorylated-(Thr180/Tyr182) p38 MAPK)	Rabbit	1:1000	Cell Signaling Technology
phosphorylated (Thr202/Tyr204) ERK1/2	Rabbit	1:1000	Cell Signaling Technology
polyclonal anti-ERK1/2	Rabbit	1:20000	Sigma Aldrich
phosphorylated (Se473) Akt	Rabbit	1:1000	Cell Signaling Technology
phosphorylated (Ser536) NF-κB(p65)	Rabbit	1:1000	Cell Signaling Technology
NF-κB(p65)	Rabbit	1:1000	Santa Cruz Biotechnology
α-actinin	Mouse	1:5000	Santa Cruz Biotechnology
α-tubulin	Mouse	1:10000	Sigma-Aldrich

Supplementary Table 3

Oligonucleotide primers for real-time PCR		
**Target Gene**	**Accession number**	**Primer Sequence (5'-3')**
Il1b	NC_000068.7	Fwd: TGACGTTCCCATTAGACAACTG<br>Rev: CCGTCTTTCATTACACAGGACA
Il4	NC_000077.6	Fwd: ATTTTGAACGAGGTCACAGGAGAAG<br>Rev: ACCTTGGAAGCCCTACAGACGAG
Il6	M_031168	Fwd: GAACAACGATGATGCACTTGC<br>Rev: CTTCATGTACTCCAGGTAGCTATGGT
Il10	NM_010548.2	Fwd: CAAGGAGCATTTGAATTCCC<br>Rev: GGCCTTGTAGACACCTTGGTC
Il10Ra	NC_000075.6	Fwd: TCATTGCATACGGGACAGAA<br>Rev: TGGATGTCATTCCAGGTTGA
Il12a	NC_000069.6	Fwd: CGCAGCACTTCAGAATCACA<br>Rev: TCTCCCACAGGAGGTTTCTG
Tnfa	NM_013693	Fwd:TCTTCTGTCTACTGAACTTCGGGGTGA<br>Rev: GTGGTTTGCTACGACGTGGGCTA
Nos2	NM_010927	Fwd: AGCCAAGCCCTCACCTACTT<br>Rev: TCTCTGCCTATCCGTCTCGT
Arg1	NM_007482	Fwd: CAATGAAGAGCTGGCTGGTGT<br>Rev: GTGTGAGCATCCACCCAAATG
Mrc1	NC_000068.7	Fwd: TCTTTGCCTTTCCCAGTCTCC<br>Rev: TGACACCCAGCGGAATTTC
Cd68	NC_000077.6	Fwd: CAAAGCTTCTGCTGTGGAAAT<br>Rev: GACTGGTCACGGTTGCAAG
Gapdh	NM_008084.2	Fwd: GCCTTCCGTGTTCCTACCC<br>Rev: CAGTGGGCCCTCAGATGC
Ifng	NM_008337.3	Fwd: GACAATCAGGCCATCAGCAAC<br>Rev: CGGATGAGCTCATTGAATGCTT
Cd86	NM_009853.1	Fwd: TTGTGTGTGTTCTGGAAACGGAG<br>Rev: AACTTAGAGGCTGTGTTGCTGGG
Cd163a	NM_053094.2	Fwd: GCAAAAACTGGCAGTGGG<br>Rev: GTCAAAATCACAGACGGAGC
Tgfb	NM_011577.1	Fwd: GAGACGGAATACAGGGCTTTC<br>Rev: TCTCGTGGAGCTGAAGCAAT
Ccl2	NC_005109.4	Fwd: GCTCAGCCAGATGCAGTTAAC<br>Rev: CTCTCTCTTGAGCTTGGTGAC
Ccr2	NC_000075.6	Fwd: CCTGTAAATGCCATGCAAGTTC<br>Rev: GTATGCCGTGGATGAACTGAG
Dursp1	NC_007125.6	Fwd: CAGATTAGGAGCAGCGAGC<br>Rev: AAAGCGAAGAAGGAGCGAC
S100b	NC_000076.6	Fwd: TGGCTGCGGAAGTTGAGATT<br>Rev: GAAGGGGGTTGGGGTTTCAT

Supplementary Results

Figure S1. Blocking S100B early after acute muscle injury delays regeneration. (**a**) S100B and S100A1 (5 μg each) were run on SDS polyacrylamide gels (15%) and either stained with blue Coomassie (top panel) or transferred onto nitrocellulose paper for western blotting using a polyclonal anti-S100B antibody (Abcam No. ab41548) (bottom panel). (**b**) C2C12 myoblasts were cultured in differentiation medium in the absence or presence of 200 ng S100B/ml ± increasing doses of a polyclonal anti-S100B antibody (Abcam No. ab41548), as indicated, and left undisturbed for 2 days. Then, cells were lysed and cell lysates were subjected to western blotting for detection of the late myogenic marker, embryonic myosin heavy chain (eMyHC) (bottom panel). Immunoblots of α-tubulin are included as loading controls. Results are means ± SEM from three experiments (upper panel). *$p<0.05$, **$p<0.01$, ***$p<0.001$ vs. control. (**c**) TA muscles were injected with $BaCl_2$ at d0, followed by injection with IgG or anti-S100B antibody at d1 p.i. Treated muscle were excised at d3 and d7 p.i. for analyses. (**d**,**e**) $PAX7^+$, MyoD+, $myogenin^+$, $Ki67^+$, and $RAGE^+$ cells were detected at d3 (d) and at d7 (e) p.i. in muscles by immunohistochemistry. The scale bar represents 50 μm in (d and e).

Figure S2. S100B affects macrophages in acutely injured muscles. (**a**) TA muscles were treated as described in the legend to Fig 2a. Muscles were excised at d3 or d7 p.i. (**b**) Macrophages were detected by MAC3 immunohistochemistry. (**c**) Immunofluorescence detection of MAC3 in macrophages isolated at d3 p.i. from muscles treated with IgG or anti-S100B antibody. (**d**) Homogenates of muscles excised at d3 or d7 p.i. were subjected to western blotting. Immunoblots of α-actinin are included as loading controls. (**e**) Double iNOS/MAC3 and CD163/MAC3 immunofluorescence at day 7 p.i. of muscles treated with IgG or anti-S100B antibody at day 1 p.i. Nuclei were counterstained with DAPI. Merged images are shown. Note in anti-S100B-treated muscles the high number of macrophages (red channel) and the co-existence of $iNOS^+$ (M1) and $CD163^+$ (M2) macrophages as opposed to the almost complete absence of M1 macrophages and the low number of M2 macrophages in IgG-treated (control) muscles. Arrows point to double $iNOS^+/MAC3^+$ (M1) and $CD163^+/MAC3^+$ (M2) macrophages. (**f**) IgG staining of injured muscles. (**g**) Peritoneal macrophages were cultured for 24 h in the absence and presence of either S100B, IFN-γ, IL-10 or IL-4 and analyzed by real-time PCR. (**h**) Macrophages isolated at d3 and d7 p.i. from injured muscles and analyzed for *S100b* levels by real-time PCR. (**i**) Detection of S100B in activated ($MAC3^+$) macrophages and in M1 ($iNOS^+$) and M2 ($CD163^+$) macrophages by double immunofluorescence in injured muscles. Nuclei were counterstained with DAPI. The scale bar represents 50 μm in b and f and 100 μm in e and i.

Figure S3. S100B regulates muscle regeneration by acting on both myoblasts and macrophages. (**a**) Mice were intraperitoneally injected with vehicle or clodronate. Injured TA muscles were injected with IgG

or anti-S100B antibody (day 1 p.i.). Treated muscle were excised at d3 p.i. (**b**) Histology of muscle tissue (upper panel) and counts of interstitial cells (lower panel). (**c**) MAC3 immunohistochemistry (upper panels) and $MAC3^+$ cell counts (lower panel). (**d**) Western blots of MAC3, CD163 and iNOS in muscle homogenates. Immunoblots of α-actinin are included as loading controls. (**e**) IgG staining. (**f**) $PAX7^+$, $MyoD^+$, $myogenin^+$ and $Ki67^+$ cell counts. (**g**) $PAX7^+$, $MyoD^+$, $myogenin^+$ and $Ki67^+$ cells were detected at d3 by immunohistochemistry. Results are means ± SEM from at least six animals. *p<0.05, **p<0.01, ***p<0.001 vs. control. ##p<0.001 (clodronate-treated vs. vehicle, b and f). The scale bar in (b, c and e) represents 50 μm.

Figure S4. S100B is required during the macrophage M2 phase for efficient regeneration. (**a**) Injured TA muscles were injected with IgG or anti-S100B antibody at d4 p.i. Treated muscle were excised at d7 or d14 p.i. (**b**) $PAX7^+$, MyoD+, $myogenin^+$, $Ki67^+$, and $MAC3^+$ cells were detected at d7 and at d14 p.i. in muscles by immunohistochemistry. (**c**) IgG staining of injured muscles and collagen IV detection by immunohistochemistry of IgG- and anti-S100B-treated injured muscles at d7. The scale bar represents 50 μm.

Figure S5. S100B's ability to promote regeneration of acutely injured skeletal muscles requires RAGE at early, but not mid-late regeneration phase. (**a**) Macrophages were isolated from wild type and *Ager*$^{-/-}$ TA muscles at d7 p.i. and analyzed for expression levels of the indicated genes by real-time PCR. Results are expressed as fold-change of gene expression levels in *Ager*$^{-/-}$ vs. wild type macrophages. (**b**) Injured *Ager*$^{-/-}$ TA muscles were injected with IgG or anti-S100B antibody at d1 p.i and excised at d3 or d5 p.i. (**c**)) Histology (upper panel) and counts of interstitial cells and centrally nucleated myofibers (lower panel). (**d**) $PAX7^+$, $MyoD^+$, $myogenin^+$, $MAC3^+$ and $Ki67^+$ cell counts. (**e**) Macrophages isolated at d3 from IgG- and anti-S100B-treated injured *Ager*$^{-/-}$ muscles and analyzed by real-time PCR. (**f**) Peritoneal macrophages from *Ager*$^{-/-}$ mice subjected to a migration assay using Boyden chambers in the presence of increasing S100B doses. (**g,h**) *Ager*$^{-/-}$ TA muscles were injected with BaCl2 at day 0, followed by injection with IgG or anti-S100B antibody at d4 p.i. and excision at d7 p.i. (g) for histology and collagen IV immunohistochemistry (h). (**i**) $PAX7^+$, $MyoD^+$, $myogenin^+$, $Ki67^+$, and $MAC3^+$ cells were detected at d7 p.i. by immunohistochemistry. Shown are representative images. The scale bar represents 50 μm.

Figure S6. Late blockade of S100B results in altered bFGF/FGFR1 signaling. (**a,b**) Conditions were as in Fig 5c (a). *Ager*$^{-/-}$ muscles were excised at d7 p.i. and analyzed for $PAX7^+$, $MyoD^+$, $myogenin^+$, $Ki67^+$, and $MAC3^+$ cells by immunohistochemistry (b). Shown are representative images. The scale bar represents 50 μm. (**c**) Same as in a except that macrophages isolated from injured muscles were analyzed for levels of

proinflammatory and antiinflammatory markers by real-time PCR. (**d**) Macrophages isolated at d4 p.i. from injured *Ager*$^{-/-}$ muscles were incubated for 30 min in the absence or presence of S100B (200 ng/ml) and lysed. Cell lysates were subjected to immunoprecipitation with anti-S100B antibody and immunoprecipitates were probed with anti-S100B and anti-FGFR1 antibodies.

Figure S7. Full-length blots of cropped blots from the manuscript (relative to Figures 1-3).

Figure S8. Full-length blots of cropped blots from the manuscript (relative to Figures 4-7).

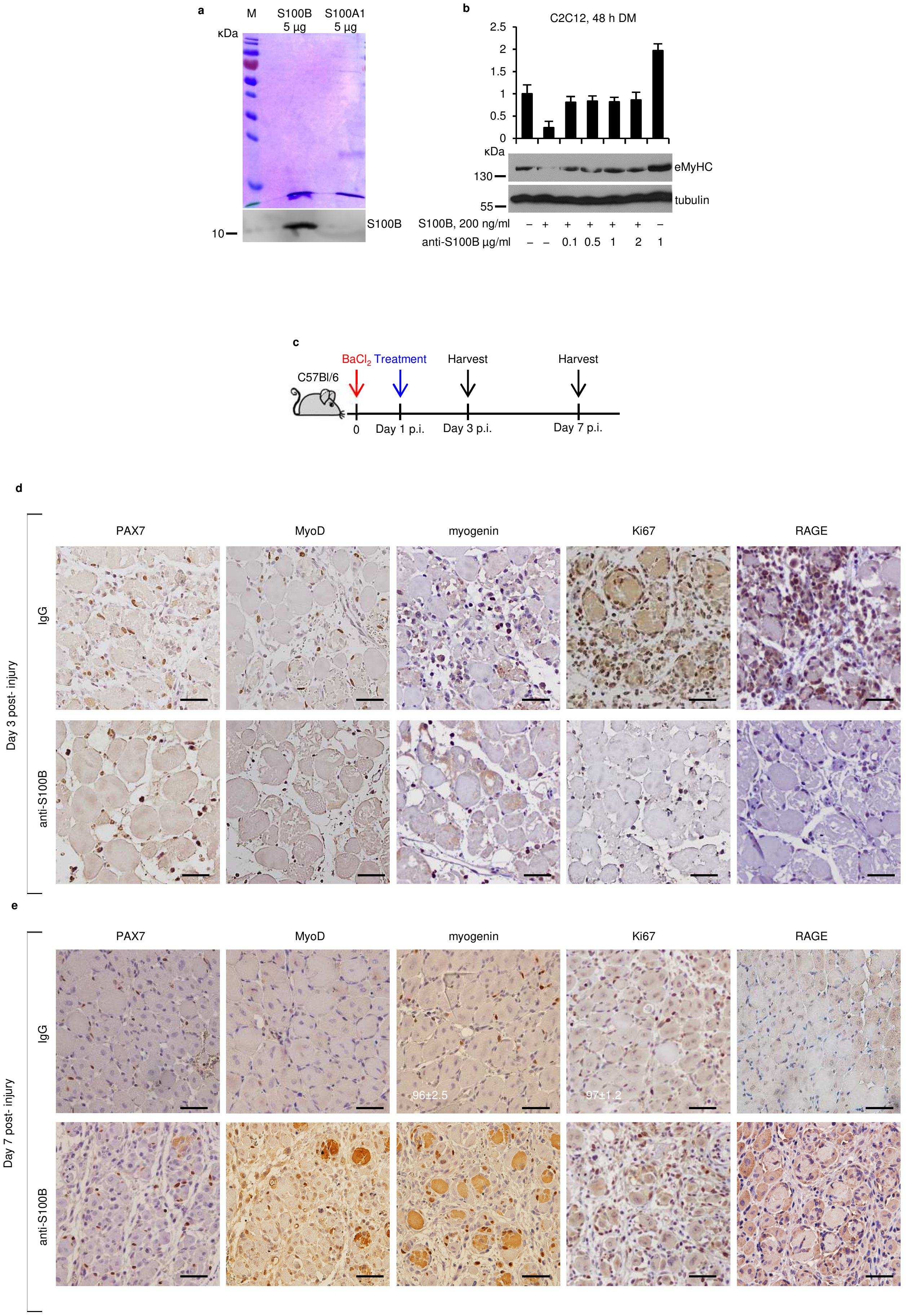

a
M
S100B 5 µg
S100A1 5 µg
κDa
10
S100B
b
C2C12, 48 h DM
2.5
2
1.5
1
0.5
0
κDa
130
55
eMyHC
tubulin
S100B, 200 ng/ml – + + + + + –
anti-S100B µg/ml – – 0.1 0.5 1 2 1
c
C57Bl/6
BaCl2
Treatment
Harvest
Harvest
0
Day 1 p.i.
Day 3 p.i.
Day 7 p.i.
d
Day 3 post- injury
IgG
anti-S100B
PAX7
MyoD
myogenin
Ki67
RAGE
e
Day 7 post- injury
IgG
anti-S100B
PAX7
MyoD
myogenin
Ki67
RAGE
96±2.5
97±1.2

Figure S1

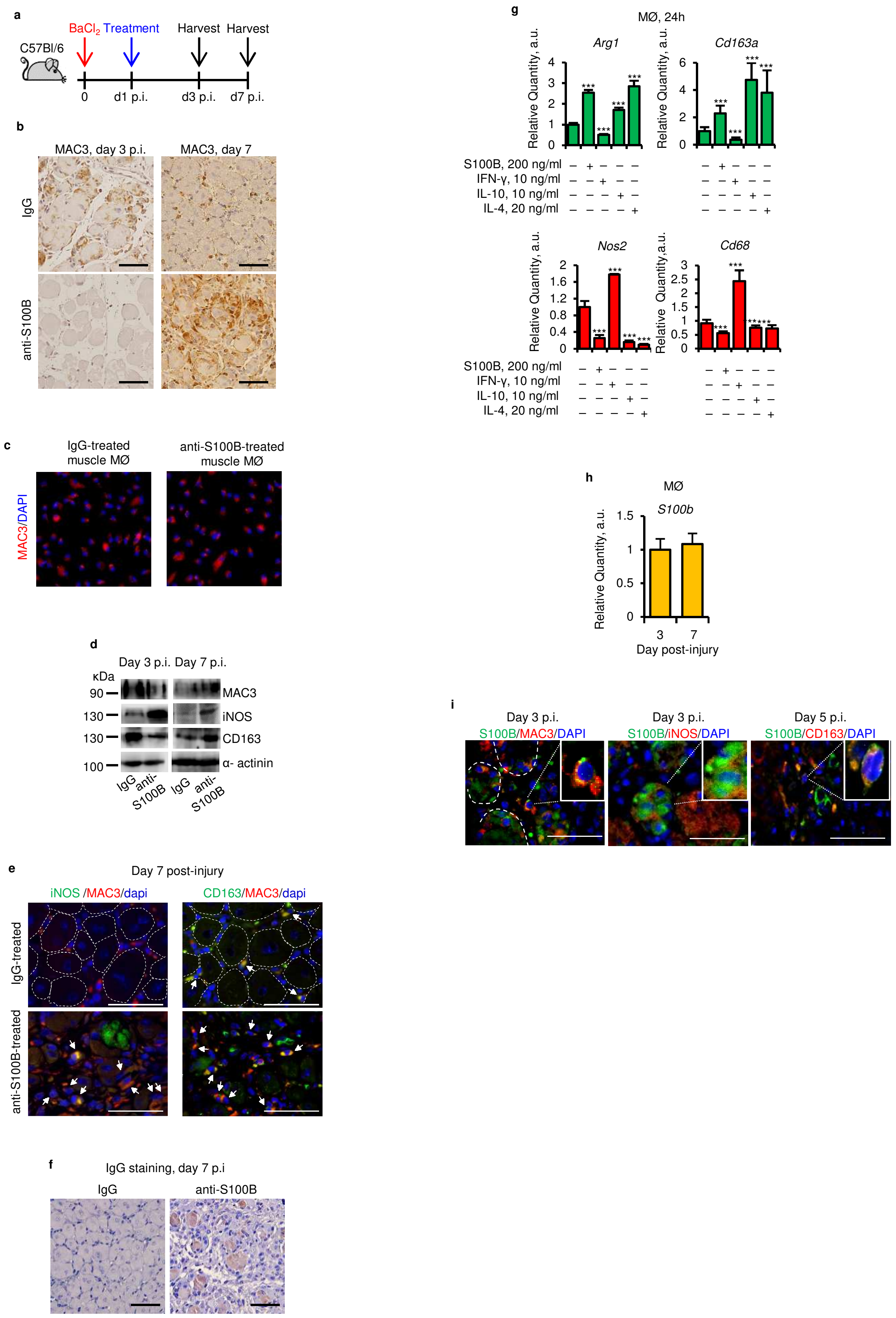
a
C57Bl/6
BaCl$_2$ Treatment
Harvest Harvest
0
d1 p.i.
d3 p.i.
d7 p.i.
b
MAC3, day 3 p.i.
MAC3, day 7
IgG
anti-S100B
c
IgG-treated muscle MØ
anti-S100B-treated muscle MØ
MAC3/DAPI
d
Day 3 p.i. Day 7 p.i.
κDa
90
130
130
100
MAC3
iNOS
CD163
α- actinin
IgG
anti-S100B
IgG
anti-S100B
e
Day 7 post-injury
iNOS /MAC3/dapi
CD163/MAC3/dapi
IgG-treated
anti-S100B-treated
f
IgG staining, day 7 p.i
IgG
anti-S100B
g
MØ, 24h
Arg1
Cd163a
Relative Quantity, a.u.
S100B, 200 ng/ml
IFN-γ, 10 ng/ml
IL-10, 10 ng/ml
IL-4, 20 ng/ml
Nos2
Cd68
h
MØ
S100b
Relative Quantity, a.u.
Day post-injury
i
Day 3 p.i.
S100B/MAC3/DAPI
Day 3 p.i.
S100B/iNOS/DAPI
Day 5 p.i.
S100B/CD163/DAPI

Figure S2

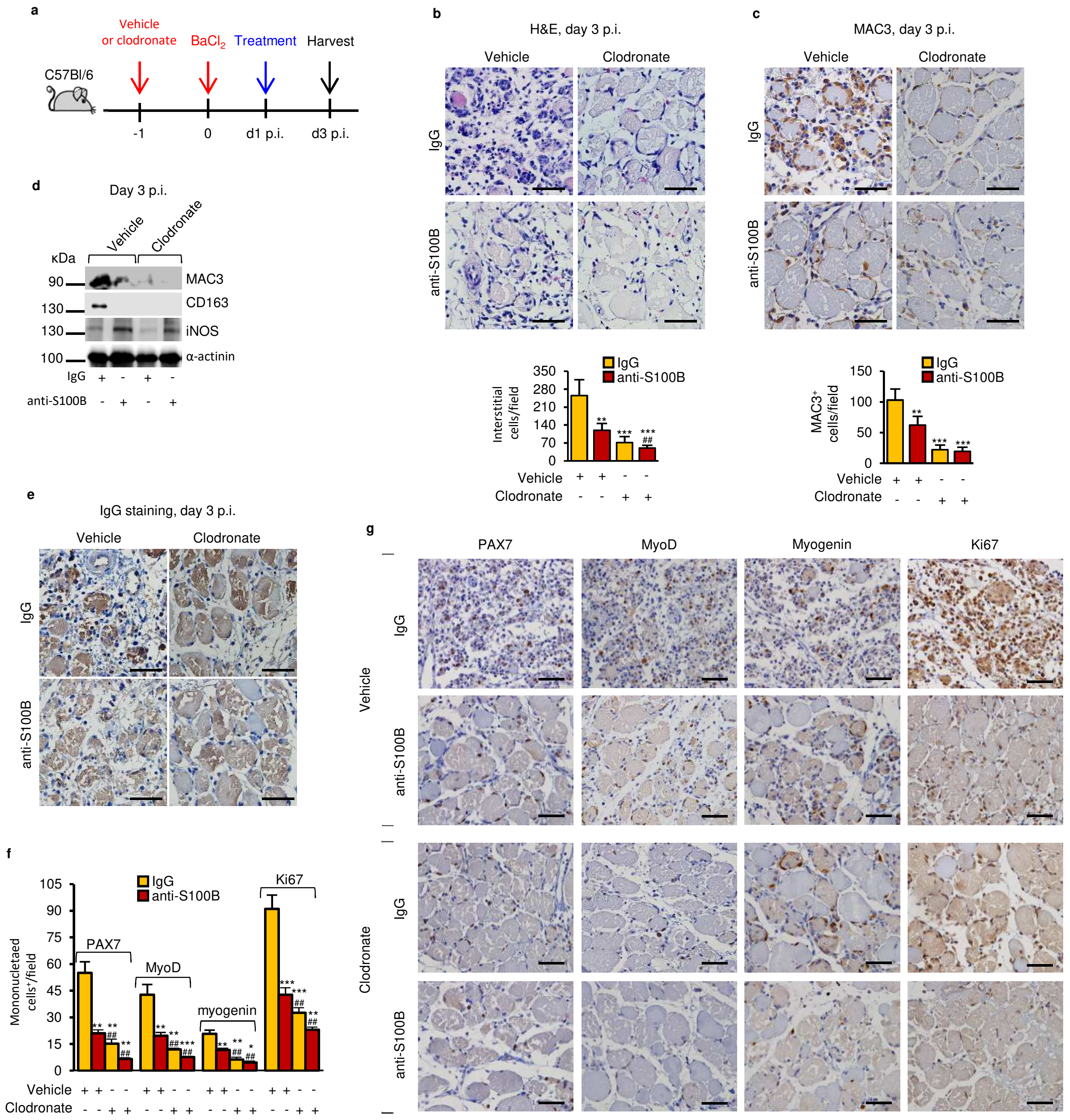
a
Vehicle or clodronate
BaCl$_2$
Treatment
Harvest
C57Bl/6
-1
0
d1 p.i.
d3 p.i.
b
H&E, day 3 p.i.
Vehicle
Clodronate
IgG
anti-S100B
Interstitial cells/field
IgG
anti-S100B
Vehicle + + - -
Clodronate - - + +
c
MAC3, day 3 p.i.
MAC3+ cells/field
d
Day 3 p.i.
κDa
MAC3
CD163
iNOS
α-actinin
anti-S100B
e
IgG staining, day 3 p.i.
f
Mononucletaed cells+/field
PAX7
MyoD
myogenin
Ki67
g
PAX7
MyoD
Myogenin
Ki67

Figure S3

a

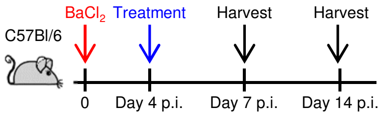

b

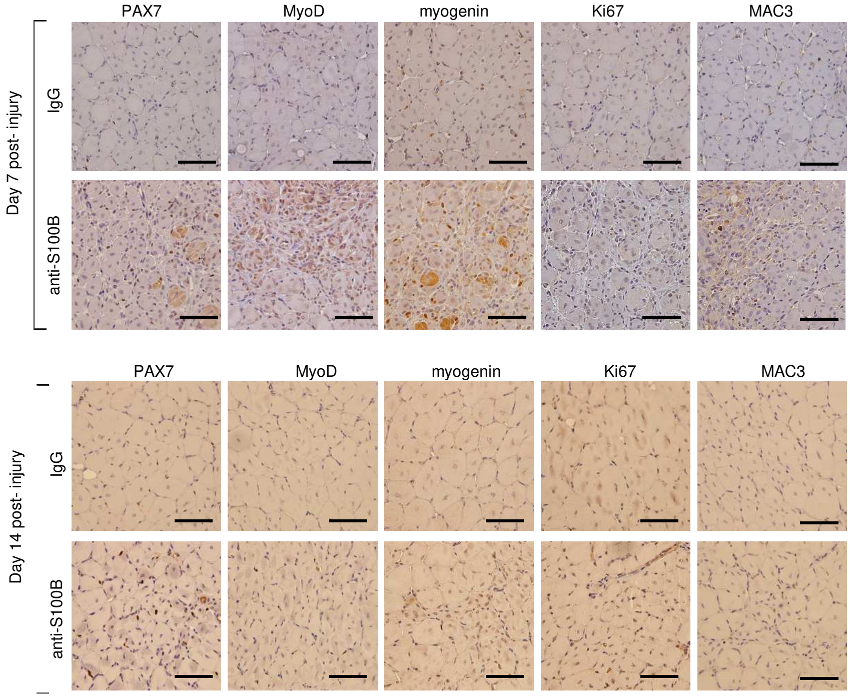

c

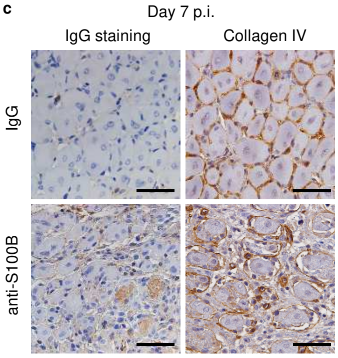

Figure S4

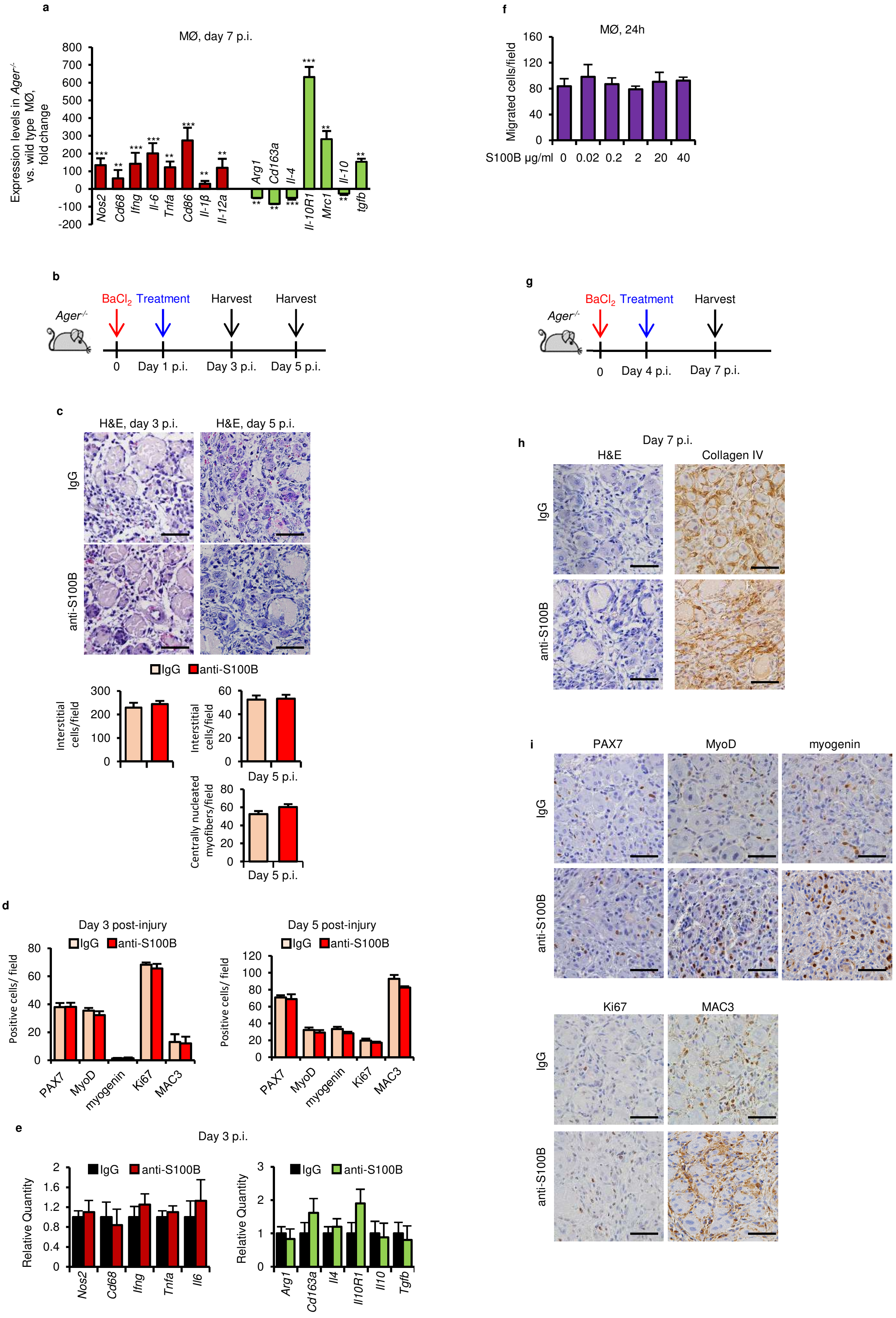

a
MØ, day 7 p.i.
Expression levels in Ager-/- vs. wild type MØ, fold change
800
700
600
500
400
300
200
100
0
-100
-200
Nos2
Cd68
Ifng
Il-6
Tnfa
Cd86
Il-1β
Il-12a
Arg1
Cd163a
Il-4
Il-10R1
Mrc1
Il-10
tgfb
f
MØ, 24h
Migrated cells/field
160
120
80
40
0
S100B µg/ml 0 0.02 0.2 2 20 40
b
Ager-/-
BaCl2 Treatment
Harvest
Harvest
0
Day 1 p.i.
Day 3 p.i.
Day 5 p.i.
g
Ager-/-
BaCl2 Treatment
Harvest
0
Day 4 p.i.
Day 7 p.i.
c
H&E, day 3 p.i.
H&E, day 5 p.i.
IgG
anti-S100B
IgG anti-S100B
Interstitial cells/field
300
200
100
0
Interstitial cells/field
60
40
20
0
Day 5 p.i.
Centrally nucleated myofibers/field
80
60
40
20
0
Day 5 p.i.
h
Day 7 p.i.
H&E
Collagen IV
IgG
anti-S100B
i
PAX7
MyoD
myogenin
IgG
anti-S100B
Ki67
MAC3
IgG
anti-S100B
d
Day 3 post-injury
IgG anti-S100B
Positive cells/ field
80
60
40
20
0
PAX7
MyoD
myogenin
Ki67
MAC3
Day 5 post-injury
IgG anti-S100B
Positive cells/ field
120
100
80
60
40
20
0
PAX7
MyoD
myogenin
Ki67
MAC3
e
Day 3 p.i.
IgG anti-S100B
Relative Quantity
2
1.6
1.2
0.8
0.4
0
Nos2
Cd68
Ifng
Tnfa
Il6
IgG anti-S100B
Relative Quantity
3
2
1
0
Arg1
Cd163a
Il4
Il10R1
Il10
Tgfb

Figure S5

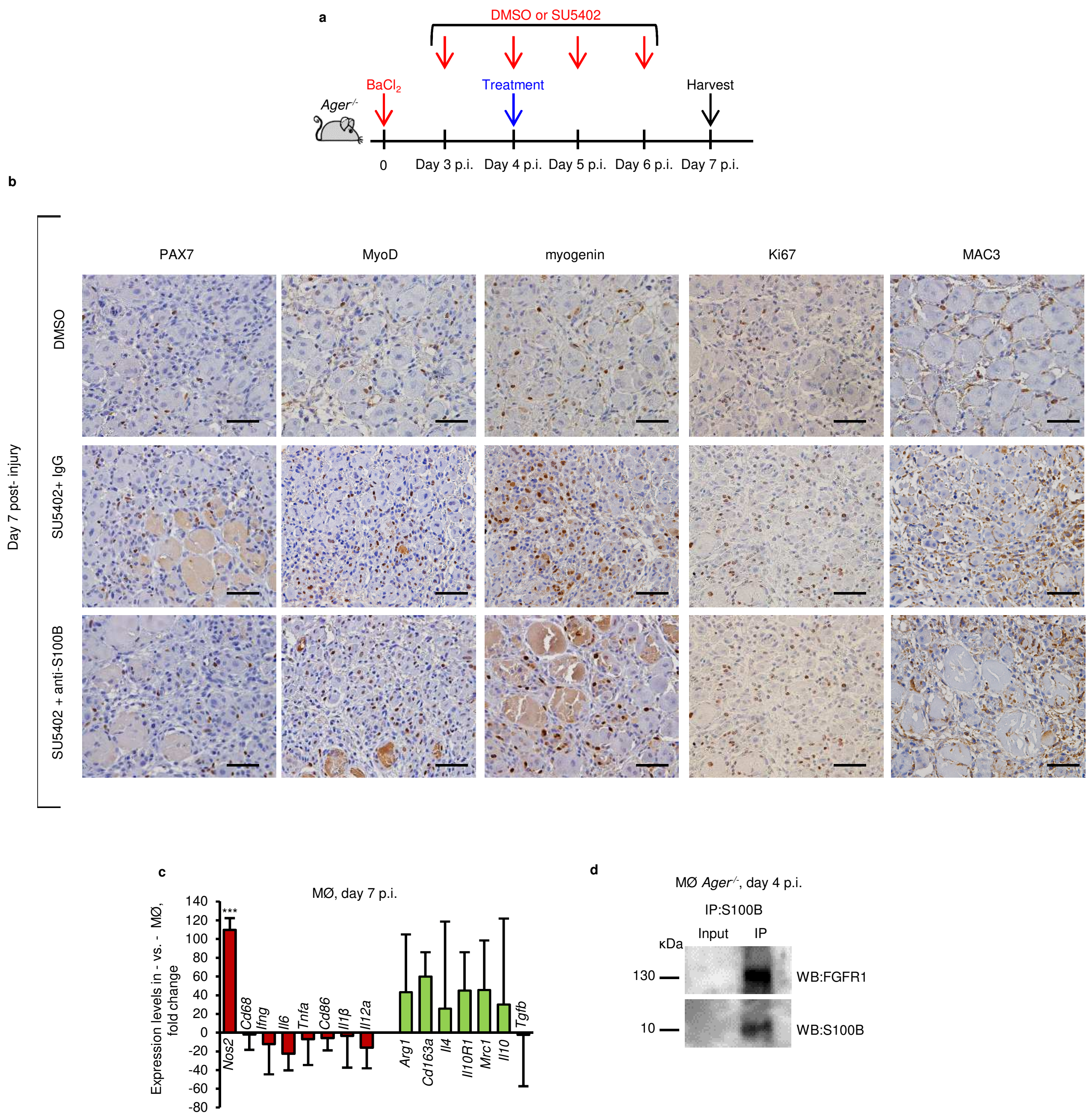
a
DMSO or SU5402
BaCl2
Treatment
Harvest
Ager-/-
0
Day 3 p.i.
Day 4 p.i.
Day 5 p.i.
Day 6 p.i.
Day 7 p.i.
b
PAX7
MyoD
myogenin
Ki67
MAC3
DMSO
SU5402+ IgG
SU5402 + anti-S100B
Day 7 post- injury
c
MØ, day 7 p.i.
Expression levels in - vs. - MØ, fold change
***
Nos2
Cd68
Ifng
Il6
Tnfa
Cd86
Il1β
Il12a
Arg1
Cd163a
Il4
Il10R1
Mrc1
Il10
Tgfb
d
MØ Ager-/-, day 4 p.i.
IP:S100B
kDa
Input
IP
130
10
WB:FGFR1
WB:S100B

Figure S6

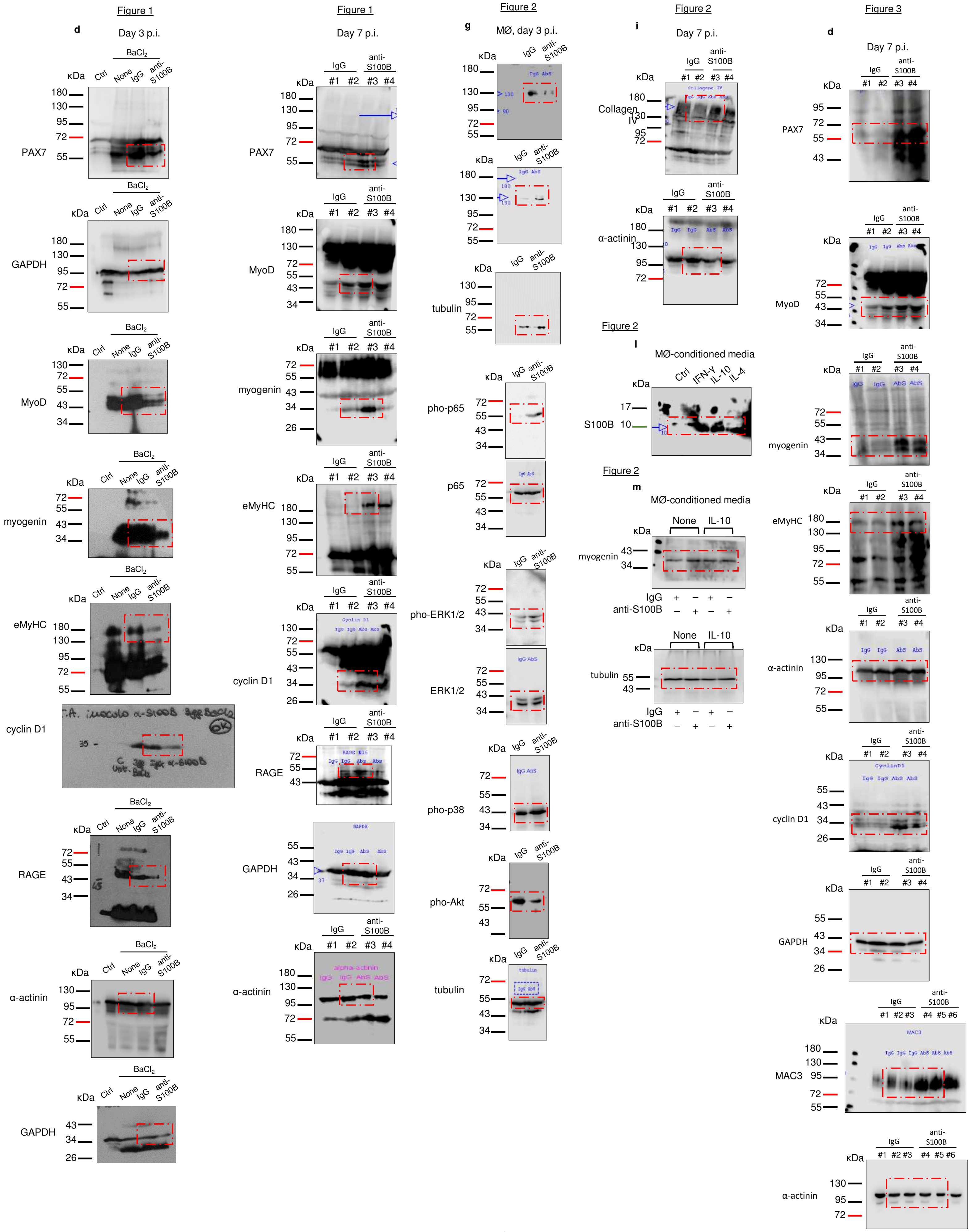

Figure 1
d
Day 3 p.i.
BaCl2
Ctrl
None
IgG
anti-S100B
kDa
PAX7
GAPDH
MyoD
myogenin
eMyHC
cyclin D1
RAGE
α-actinin
Figure 1
Day 7 p.i.
IgG
anti-S100B
#1 #2 #3 #4
PAX7
MyoD
myogenin
eMyHC
cyclin D1
RAGE
GAPDH
α-actinin
Figure 2
g
MØ, day 3 p.i.
tubulin
pho-p65
p65
pho-ERK1/2
ERK1/2
pho-p38
pho-Akt
tubulin
Figure 2
i
Day 7 p.i.
Collagen IV
α-actinin
Figure 2
l
MØ-conditioned media
Ctrl
IFN-γ
IL-10
IL-4
S100B
Figure 2
m
MØ-conditioned media
None
IL-10
myogenin
IgG + − + −
anti-S100B − + − +
tubulin
Figure 3
d
Day 7 p.i.
PAX7
MyoD
myogenin
eMyHC
α-actinin
cyclin D1
GAPDH
#1 #2 #3 #4 #5 #6
MAC3
α-actinin

Figure S7

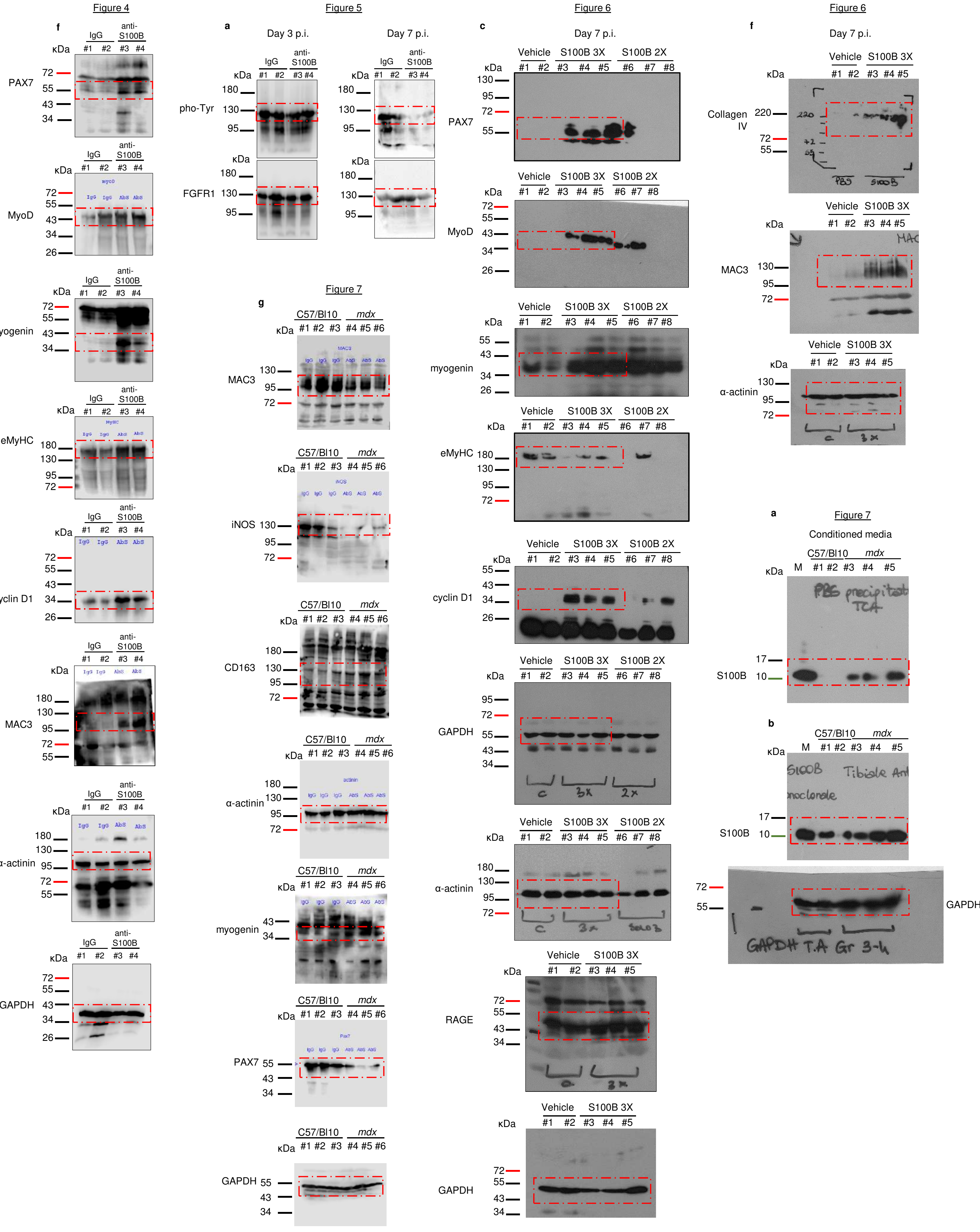
Figure 4
f
IgG
anti-S100B
#1 #2 #3 #4
kDa
PAX7
MyoD
myogenin
eMyHC
cyclin D1
MAC3
α-actinin
GAPDH
72
55
43
34
26
180
130
95
Figure 5
a
Day 3 p.i.
Day 7 p.i.
pho-Tyr
FGFR1
Figure 7
g
C57/Bl10
mdx
#1 #2 #3 #4 #5 #6
MAC3
iNOS
CD163
α-actinin
myogenin
PAX7
GAPDH
Figure 6
c
Day 7 p.i.
Vehicle
S100B 3X
S100B 2X
#1 #2 #3 #4 #5 #6 #7 #8
PAX7
MyoD
myogenin
eMyHC
cyclin D1
GAPDH
α-actinin
RAGE
Figure 6
f
Day 7 p.i.
Vehicle
S100B 3X
#1 #2 #3 #4 #5
Collagen IV
220
MAC3
α-actinin
Figure 7
a
Conditioned media
C57/Bl10
mdx
M #1 #2 #3 #4 #5
S100B
17
10
b
GAPDH

Figure S8
